# Sex-specific effects of alcohol on neurobehavioral performance and endoplasmic reticulum stress: an analysis using neuron-specific MANF deficient mice

**DOI:** 10.3389/fphar.2024.1407576

**Published:** 2024-07-26

**Authors:** Wen Wen, Hui Li, Marisol Lauffer, Di Hu, Zuohui Zhang, Hong Lin, Yongchao Wang, Mariah Leidinger, Jia Luo

**Affiliations:** ^1^ Department of Pathology, University of Iowa Carver College of Medicine, Iowa City, IA, United States; ^2^ Neural Circuits and Behavior Core, University of Iowa Carver College of Medicine, Iowa City, IA, United States; ^3^ Vanderbilt Memory and Alzheimer’s Center, Department of Neurology, Vanderbilt University Medical Center, Nashville, TN, United States; ^4^ Comparative Pathology Laboratory, University of Iowa Carver College of Medicine, Iowa City, IA, United States; ^5^ Iowa City VA Health Care System, Iowa City, IA, United States

**Keywords:** excessive alcohol exposure, MANF, neurobehavioral deficits, ER stress, neuroinflammation

## Abstract

Excessive alcohol exposure can cause neurobehavioral deficits and structural alterations in the brain. Emerging research evidence suggests that endoplasmic reticulum (ER) stress plays an important role in alcohol-induced neurotoxicity. Mesencephalic astrocyte-derived neurotrophic factor (MANF) is an ER stress inducible protein and is responsible to maintain ER homeostasis. MANF is highly expressed in both the developing and mature brain. We have previously shown that MANF deficiency exacerbated alcohol induced neurodegeneration and ER stress in the developing brain. However, little is known regarding the role of MANF in alcohol induced neuronal damage in the adult brain. In this study, we used a neuron-specific MANF knockout (KO) mouse model to investigate the effect of MANF deficiency on acute binge alcohol exposure-induced neurobehavioral deficits and ER stress. Adult male and female MANF KO mice and littermate controls received daily alcohol gavage (5 g/kg) for 10 days and then subjected to a battery of neurobehavioral tests including rotarods, balance beam, DigiGait, open field, elevated plus maze, Barnes maze, and three-chamber sociability task. Female MANF KO animals were more susceptible to alcohol-induced body weight loss. Alcohol exposure did not affect motor function, however female but not male MANF KO mice exhibited an increased locomotor activity in open field test. Learning and memory was not significantly impaired, but it was altered by MANF deficiency in females while it was affected by alcohol treatment in males. Both alcohol-exposed male and female MANF KO mice displayed increased sociability. Alcohol induced the expression of ER chaperones GRP78 and GRP94 and altered the levels of several unfolded protein response (UPR) and neuroinflammation markers in MANF KO mice in a sex-specific manner. The expression of MANF interacting proteins neuroplastin, PDIA1, and PDIA6 was increased in MANF KO mice, and was further induced by alcohol. In conclusion, alcohol exposure and neuronal MANF deficiency interacted to alter neurobehavioral outcomes, ER homeostasis and neuroinflammation in a sex-specific manner.

## Introduction

Excessive alcohol use includes binge drinking (5 or more drinks for men, or four or more drinks for women, on the same occasion), heavy drinking (binge drinking on five or more days in the past month), and any alcohol use by people younger than 21 years old or pregnant women. It is a major challenge to public health worldwide and is the leading cause of preventable death (Rehm et al., 2009; GBD-2016-Alcohol-Collaborators, 2018; Slater and Alpert, 2023). Clinical and experimental studies have demonstrated that excessive alcohol exposure has adverse effects systematically, especially on the central nervous system (CNS). In both adult and developing brain, excessive alcohol exposure usually lead to disrupted cellular and molecular homeostasis, functional impairments, and even neurodegeneration that have short- and long-term cognitive and behavioral consequences (Ward et al., 2009; Alfonso-Loeches and Guerri, 2011; Hermens et al., 2013; Le Berre et al., 2014). The developing brain is vulnerable to the alcohol neurotoxicity. Prenatal alcohol exposure during pregnancy is the leading cause of preventable intellectual disabilities in the US (May et al., 2014; Brancato et al., 2020). It can cause a wide range of long-lasting physiological and neurocognitive impairments, collectively referred to as fetal alcohol spectrum disorders (FASD) (Wilhoit et al., 2017). It also leads to the inheritance of mood disturbances and increased vulnerability to alcohol in adolescent offspring (Bolstad et al., 2022). Additionally, binge drinking during adolescence is associated with alterations in psychological profiles (Das et al., 2016; Brancato et al., 2021; Castelli et al., 2022; Tringali et al., 2023). In the adult brain, reduced volumes in cortical and subcortical regions were found in people with heavy alcohol use and alcohol use disorder (AUD), with the frontal cortex being the most significantly affected (Kubota et al., 2001; Harper and Matsumoto, 2005). Both chronic alcohol abuse and acute binge drinking is associated with neurological deficits in motor function, decision making, emotion and aggressiveness, social interacting, and learning and memory (Ornelas et al., 2015; Raymond et al., 2019; Karlsson et al., 2022; Sánchez-Marín et al., 2022; Spinola et al., 2022).

Despite decades of research, the understanding of the mechanisms underlining alcohol neurotoxicity is still incomplete. Several potential mechanisms have been proposed; these include neuroinflammation, oxidative stress, and alterations in neurotransmitters and neurotrophic factors (Luo and Miller, 1998; Hernández et al., 2016; Boschen and Klintsova, 2017; Pascual et al., 2021). Recently, increasing evidence suggested that endoplasmic reticulum (ER) stress plays an important role in alcohol-induced CNS damages (Yang and Luo, 2015; Wang et al., 2018). Alcohol exposure has been shown to cause ER stress in various organs, such as the liver, pancreas, muscle, heart, and the CNS (Pandol et al., 2010; Ji, 2012; Yang and Luo, 2015). The ER is an important organelle that regulates protein folding and secretion. Correctly folded proteins exit the ER and are transported to the Golgi for secretion. Misfolded/unfolded proteins, however, are retained and accumulated in the ER, triggering ER stress. A conserved protective machinery called unfolded protein response (UPR) is activated in response to ER stress. UPR is mediated by three ER transmembrane proteins: pancreatic ER kinase-like ER kinase (PERK), inositol-requiring enzyme 1 α (IRE1α), and activating transcription factor 6 (ATF6) (Walter and Ron, 2011). The activation of UPR results in increased ER chaperone expression that facilitates protein folding, temporary attenuation of translation that slows down new protein synthesis, and activation of ER-associated protein degradation (ERAD) that removes aberrant proteins (Hetz and Saxena, 2017). ER stress is usually resolved by UPR. However, prolonged ER stress and failed attempts to achieve ER homeostasis can ultimately result in apoptosis (Xu et al., 2005; Rasheva and Domingos, 2009). We and other investigators have shown that alcohol induces ER stress in both the developing and mature brain (Dlugos, 2006; Ke et al., 2011; Dlugos, 2014; George et al., 2018; Wang et al., 2018; Li et al., 2019; Xu et al., 2019; Xu et al., 2021). Alcohol exposure during developmental stages in the third trimester equivalent mouse model upregulates several UPR genes in the postnatal day (PD) seven mouse brain, including GRP78, CHOP, XBP1s, and ATF6 (Wang et al., 2021). Immature neurons are more susceptible to alcohol neurotoxicity due to inefficient UPR machinery (Alimov et al., 2013; Li et al., 2019). Pharmacological inhibition of ER stress protects the alcohol-sensitive immature neurons from alcohol-induced neurodegeneration in the developing brain (Li et al., 2019). ER stress was also observed in the adult brain with either acute binge alcohol exposure or chronic alcohol abuse (Wang et al., 2018; Xu et al., 2019; Xu et al., 2021). However, it is unknown whether ER stress contributes to alcohol-induced neurotoxicity and neurobehavioral deficits in adult animals.

Mesencephalic astrocyte-derived neurotrophic factor (MANF), also known as arginine-rich, mutated in early-stage tumors (ARMET), is an ER resident protein with cytoprotective functions (Petrova et al., 2003; Mizobuchi et al., 2007). The expression and secretion of MANF is induced by ER stress, which functions to maintain ER homeostasis (Wen et al., 2023). Evidence indicates that MANF alleviates ER stress-induced neuronal damages in various neurodegenerative diseases and neuronal injuries (Apostolou et al., 2008; Airavaara et al., 2009; Neves et al., 2016). The mechanism of MANF’s neurotrophic function is unclear, and it is suggested through the maintenance of ER homeostasis and immune modulation (Neves et al., 2020; Kovaleva et al., 2023; Sousa et al., 2023). MANF is strongly expressed in neurons in the developing and mature brain, and is involved in the regulation of neurogenesis, neuronal migration, and neurite outgrowth during neuronal development (Tseng et al., 2017a; Tseng et al., 2017b; Wen et al., 2020; Wang et al., 2022). We have demonstrated that subcutaneous alcohol injection (1.5 g/kg) at PD 7, which was equivalent to the third trimester of human pregnancy, can induce ER stress and MANF expression in the developing mouse brain (Ke et al., 2011; Wang et al., 2021). The upregulation of MANF expression protect against alcohol neurotoxicity in both neuronal cell cultures and postnatal mouse brains (Wen et al., 2023). Loss of neuronal MANF exacerbated alcohol-induced neurodegeneration in the immature brain (Wang et al., 2021). However, the role of MANF and ER stress in alcohol neurotoxicity in the adult brain is unknown. Using a neuron-specific MANF KO mouse model, we aimed to determine if neuronal MANF deficiency contribute to acute binge alcohol-induced neurobehavioral deficits and ER stress in adult animals. Given the unique function of MANF in ER homeostasis, this study sought to gain insight into the role of ER stress in alcohol’s effects on adult brain. In this study, we evaluated alcohol-induced neurobehavioral deficits in control and neuron specific MANF deficient adult mice through a battery of neurobehavioral tests and examined the effects of alcohol on ER stress, neuroinflammation, as well as the expression of MANF interacting proteins in the brain.

## Materials and methods

### Reagents

Ketamine/xylazine was obtained from Butler Schein Animal Health (Dublin, OH). Ethanol 200 proof (2,716) was obtained from Decon Labs, Inc. (King of Prussia, PA). Fast Tissue/Tail PCR Genotyping Kit (G1001) was obtained from EZ BioResearch (St. Louis, MO). DC protein assay Kit II (5000112) was obtained from Bio-Rad (Hercules, CA). ECL Prime Western Blotting Detection Reagent (45-002-401) was obtained from GE Healthcare Life Sciences (Piscataway, NJ).

The following antibodies were used for this study: anti-ARMET/ARP (MANF) (ab67271), anti-ATF6 (ab203119), anti-phosphorylated IRE1α (ab48187), and anti-PDIA1/P4HB (ab2792) were from Abcam (Cambridge, MA). Anti-GRP78 was from Novus Biologicals (Littleton, CO). Anti-GRP94 (ADI-SPA-850) was from Enzo Life Sciences (Farmingdale, NY). Anti-β-Actin (3,700), anti-phosphorylated PERK (3,179), anti-PERK (3,192), anti-phosphorylated eIF2α (3,398), anti-eIF2α (9,722), anti-ATF4 (11,815), anti-IRE1α (3,294), anti-cleaved caspase-3 (9,661), and anti-IL6 (12,912) were from Cell Signaling Technology (Danvers, MA). Anti-XBP1s (658,802) was from BioLegend (San Diego, CA). Anti-CHOP (MA1-250) was from Thermo Fisher Scientific (Rockford, IL). Anti-MCP1 (AAM-43) was from Bio-Rad (Hercules, CA). Anti-CCR2 (3415R) was from BioVision. Anti-CYP2E1 (MA5-32605), anti-Catalase (PA5-29183), anti-ALDH1A1 (15910-1-AP), and anti-ALDH2 (PA5-27414) were from Invitrogen (Waltham, MA). Anti-Neuroplastin (AF7818) was from R&D Systems (Minneapolis, MN). Anti-PDIA6 (18233-1-AP) was from Proteintech (Rosemont, IL). HRP-conjugated anti-rabbit (GENA934) and anti-mouse (GENA931) secondary antibodies were from GE Healthcare Life Sciences (Piscataway, NJ).

### Animals

All experimental animal procedures were approved by the Institutional Animal Care and Use Committee (IACUC) at the University of Iowa (#3042295) and performed following regulations for the Care and Use of Laboratory Animals set forth by the National Institutes of Health (NIH) Guide. Animals used in this study were group housed with a maximum of five animals per cage in clear cage with filter top lid. Animals were allowed *ad libitum* access to chow and water on a 12-h light/12-h dark cycle. Neuron-specific MANF KO mice was generated and described previously (Lindahl et al., 2014; Pakarinen et al., 2020; Wang et al., 2021). Briefly, mouse embryonic stem cell clone EPD0162_3_D06 Manf ^tm1a(KOMP)Wtsi^ (C57BL/6N origin) was generated by the Wellcome Trust Sanger Institute (WTSI) (Testa et al., 2004). It was microinjected into the blastocyte using Balb/c donors by the Knockout Mouse Project (KOMP) Repository. Resulting chimeras were mated to C57BL/6N mice and heterozygous animals carrying the Manf ^tm1a(KOMP)Wtsi^ allele were generated. Through the Mouse Biology Program (MBP) at the University of California Davis, they were crossed two generations with the FLP recombinase line C57BL/6-Tg (CAG-Flpo)1Afst/Mmucd (032247-UCD) obtained from the Mutant Mouse Regional Resource Center (MMRRC) to ensure excision in the germ cells and removal of the FLP transgene. The resulting mice were bred to homozygosity and referred to as *Manf* ^fl/fl^ and thereafter. To achieve neuron-specific MANF KO, *Manf* ^fl/fl^ mice were crossed with *Nestin* ^Cre+/−^ transgenic mice B6.Cg-Tg(Nes-Cre)1Kln/J purchased from Jackson Laboratory, as described previously (Wang et al., 2021). The *Nestin* ^Cre+/−^ transgenic mice drive Cre-Lox recombination in neural stem cells and intermediate neural progenitor cells as early as embryonic day 12.5 and a gradually increased recombination was observed during perinatal stages (Tronche et al., 1999; Dubois et al., 2006; Liang et al., 2012). As a result, the adult *Manf* ^fl/fl^::*Nestin* ^Cre+/−^ mice are considered neuron-specific MANF KO. All mice used in this study were from further crossing of *Manf* ^fl/fl^ mice with *Manf* ^fl/fl^::*Nestin* ^Cre+/−^ mice that produce theoretically half of the offspring as *Manf* ^fl/fl^::*Nestin* ^Cre+/−^ (referred to as MANF KO), and half as *Manf* ^fl/fl^ (referred to as littermate controls). All mice were genotyped by polymerase chain reaction (PCR) analysis using genomic DNA extracted from tail clippings by the Fast Tissue/Tail PCR Genotyping Kit (G1001, EZ BioResearch) according to the manufacturer’s instructions. Primers used for genotyping were ordered from Integrated DNA Technologies (Coralville, IA) and as follows: Manf (f) 5′- TGA​AGC​AAG​AGG​CAA​AGA​GAA​TCG​G-3′, Manf (r) 5′- TGC​TCA​GCT​GCA​GAG​TTA​GAG​TTC​C-3′, Cre (f) 5′-GGT​TCG​CAA​GAA​CCT​GAT​GG-3′, Cre (r) 5′-GCC​TTC​TCT​ACA​CCT​GCG​G-3′. Manf f/r generates a 511-base pair (bp) produce for wildtype allele (+), and a 718 bp product for floxed allele (fl). Cre f/r generates a 570 bp product in Cre hemizygous individual and no product for Cre negative individual. PCR products were visualized by electrophoresis on 1% agarose gel stained with ethidium bromide. KO animals were housed separately with control littermates.

### Alcohol administration

**FIGURE 1:**
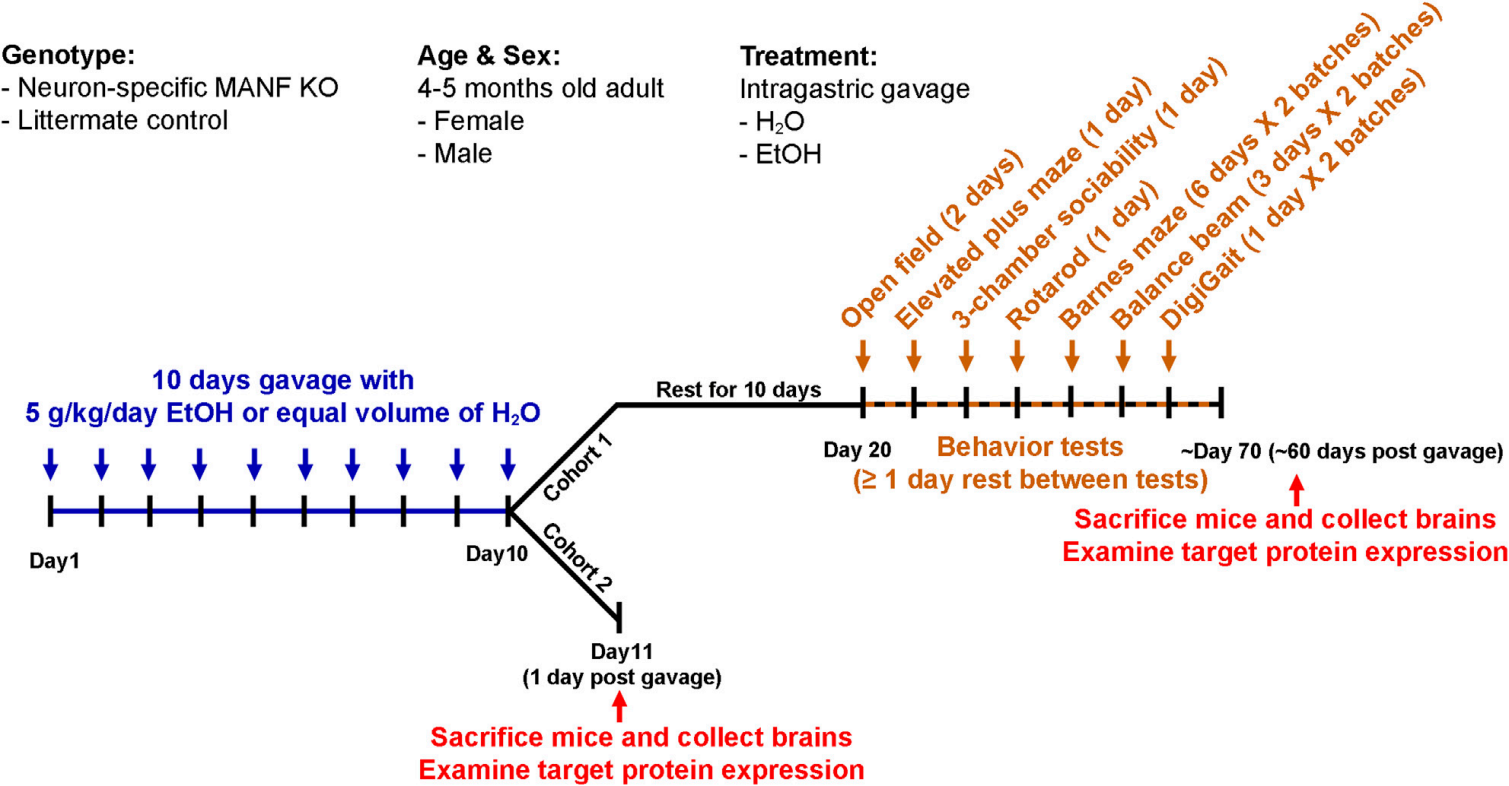
Experimental timeline. Schematic illustration of the experimental timeline of cohort 1 and cohort 2. Both male and female adult control and MANF KO mice at the age of four to five months old received equal volume of water or alcohol (5 g/kg, 25% ethanol w/v) *via* intragastric gavage once daily for 10 days. Cohort 1: animals were rested for 10 days and subjected to a battery of behavioral tests. At least 1 day rest was given between tests. Animals were randomly divided into two batches for Barnes maze, balance beam, and DigiGait tests. After behavioral tests, brain tissues were collected for neurochemical analysis. Cohort 2: animals were sacrificed 1 day after the last gavage and brain tissues were used for neurochemical analysis.

An acute binge-like alcohol exposure model was used for this study (Carson and Pruett, 1996; Karelina et al., 2017). Both male and female adult mice at the age of four to 5 months old were randomly assigned to H_2_O or ethanol groups. Animals were housed in separate cages by treatment. They received equal volume of H_2_O or ethanol (5 g/kg, 25% ethanol w/v) *via* intragastric gavage once daily for 10 days and then subjected to behavioral tests and molecular analysis as described below. This alcohol exposure paradigm has been widely used to investigate alcohol-induced neurodegeneration, glial cell activation and neuroinflammation, and traumatic brain injury in adult and adolescent mice (Carson and Pruett, 1996; Nixon and Crews, 2002; Crabbe et al., 2011; Bertola et al., 2013; Kane et al., 2014; Karelina et al., 2017; Wang et al., 2018). The blood alcohol concentration (BAC) using this paradigm peaks above 300 mg/dl 1 hour after the last ethanol administration (Wang et al., 2018), which was not rare in binge drinkers in humans (Lindblad and Olsson, 1976; Jones and Harding, 2013). All mice were given *ad libitum* access to chow and water throughout the 10 days of alcohol administration. Two cohorts of animals underwent this paradigm of acute alcohol binge exposure. Animals in cohort 1 were allowed to rest in their home cage for 10 days then followed with a battery of behavior tests to examine the effects of alcohol exposure and neuronal MANF deficiency on neurobehaviors. After behavioral tests, which was about 60 days after the last gavage, all cohort 1 animals were sacrificed, and brain tissues were used for immunoblotting analysis of target proteins. To capture the time window of molecular changes in the brain shortly after alcohol exposure, a second cohort of animals were sacrificed 1 day after the last gavage. The timeline for alcohol exposure, behavioral tests, and tissue collection were illustrated in Figure 1.

### Behavioral tests

A total of 68 animals were used for behavioral tests, with 6–10 animals in each treatment group. The exact number of mice in each experiment group were as follows: for female, control H_2_O n = 9, control ethanol n = 10, KO H_2_O n = 8, KO ethanol n = 8; for male, control H_2_O n = 9, control ethanol n = 6, KO H_2_O n = 9, KO ethanol n = 9. Tests of rotarods, balance beam, and DigiGait were performed to evaluate motor functions. Tests of open field and elevated plus maze were performed to evaluate anxiety-like behaviors. Barnes maze test was performed to evaluate learning and memory. Three-chamber sociability task was performed to evaluate social behaviors. All tests were performed at the University Iowa Neural Circuits and Behavior Core (NCBC). Behavioral tests were started 10 days after the last alcohol exposure. Animals were given at least a 1-day rest in between tests to decrease carryover effects from prior tests. Due to the limited maximum numbers of animals that can be tested in 1 day, animals were randomly divided into two batches and tested separately for balance beam, DigiGait, and Barnes maze tests. The order of tests was as follows: Day 1–2, open field; Day 5, elevated plus maze; Day 8, three-chamber sociability task; Day 10, rotarod; Day 21–26, Barnes maze for batch 1; Day 29–34, Barnes maze for batch 2; Day 29–31, balance beam for batch 1; Day 33, DigiGait for batch 1; Day 36–38, balance beam for batch 2; Day 40, DigiGait for batch 2.

### Balance beam

Balance beam test (Conduct Science MazeEngineers, Skokie, IL) was used to assess motor coordination. An 80 cm-long beam is elevated 50 cm from the floor. A safe zone is attached to one end of the beam, containing clean bedding and food. Two different beam diameters were used: 12 mm and 6 mm. Mice behavior were recorded using a front camera and an overhead camera. Each animal was trained for two consecutive days to cross the beam three trials per beam width each day, and then tested for 1 day. During training day 1, if an animal stopped crossing the beam, its tail was gently prompt to encourage movement. No prompting was performed during training day 2 and on the testing day. If an animal fell from the beam, it was placed back on the end of the beam to restart crossing. During the test day, mice were first tested on the 12 mm diameter beam and then the 6 mm diameter beam. The latency to cross the beam were recorded. Each animal was tested for three trials per beam width with 5–10 min of rest between trials. Any pauses, stalls, prompts, assists, falls, or reversals were recorded. A pause is defined as the mouse pauses for less than 5 s and then proceeds without prompting. A stall is defined as the mouse pauses for more than 5 s and then proceeds without prompting. A trial with stall was invalid and cannot be scored for the latency to cross. A trial was also invalid if the mouse falls or stalls for more than 5 s and will not proceed without prompting.

### Rotarod

Motor activity of mice was tested with the accelerating rotarod (IITC Life Science, Woodland Hills, CA). For the training trial, mice were placed on the rotarod with a constant speed of four revolutions per minute (rpm) for 5 min. For the testing trial, the rotarod was set to accelerate from 4 to 40 rpm over the course of 5 min. The latency to fall was digitally recorded. Each mouse was tested for three testing trials with 10–15 min to rest in between trials and the average latency to fall was calculated.

### DigiGait

Gait profile was measured using the DigiGait Imaging System (Mouse Specifics, Framingham, MA). Each mouse was tested on three speeds: 10 cm/s, 20 cm/s, and 30 cm/s. Animal was acclimated in the treadmill compartment for 1 min and then treadmill was turned on at a low speed (5 cm/s) and gradually increased to the testing speed over several seconds. Videos of the mice walked on the DigiGait treadmill were recorded by a high-speed video camera positioned below the transparent belt. Animals that were unable to walk at the target speed were removed from the study. Videos with at least 10 consecutive strides within 5 s were analyzed using the DigiGait software. Both spatial and temporal parameters were measured. Spatial parameters include paw area, step angle, stance width, and stride length; temporal parameters include stride time, swing time, braking time, and propulsion time.

### Open field

All animals were habituated in the testing room at least 30 min before testing starts. Each mouse was placed in the center of a custom made chamber (16″×16.5″×12″) with opaque black walls and white floor. The center of the open field was defined as the central zone with a length and width half the length and width of the chamber. All testing was conducted under 110–130 lux of lighting across the chamber. Activity was recorded for 10 min for each animal once a day for two consecutive days to look at anxiety/locomotion over time. Videos were analyzed using EthoVision XT 17 system (Noldus Information Technology, Wageningen, Netherlands). The total distance traveled, and time spent in the center of the open field were calculated.

### Elevated plus maze

All animals were habituated in the testing room at least 30 min before testing starts. Anxiety-like behavior was tested using the elevated plus maze (San Diego Instruments, San Diego, CA). The testing room was luminated by an 18″ ring light (Inkeltech) mounted around overhead camera that generates 215 lux of light in the open arms. Each animal was placed in the central area then activity was recorded for 5 min. The time spent and number of entries into the open/closed arms were analyzed by the EthoVision XT 17 system.

### Barnes maze

Learning and memory was tested using the Barnes maze test. The Barnes maze is a white circular platform (36″ diameter) elevated 40″ from the floor with 20 holes (2″ diameter) that are equally distributed around the perimeter edge (San Diego Instruments w/Custom Legs, San Diego, CA). The maze was divided into four quadrants consisting of five holes each. The target quadrant is the one with the target hole in the center. The testing room was luminated with 1,300 lux of light. The test lasted for 6 days. Day 0 was for all animals to habituate on the maze. An escape box was placed under one of the holes that was not to be used during training/acquisition and probe testing trials. All other holes were blocked. Each mouse was placed on the center of the maze and covered with the opaque start box. After 10 s, the start box was removed to allow the mouse to explore the maze freely. After 1 min, mouse was gently directed towards/into the escape box. Day 1–4 were for training trials to test the mice’s ability in learning. There were four visual cues present on the walls and the escape hole location was rotated between mice. Each mouse was placed on the center of the maze and explore the maze to find and enter the escape box. The trial ended when the mouse entered the escape box or 3 min have elapsed. Training was conducted four times per day for four consecutive days. Mice were rested 10–15 min between trials. On day 5 (24 h after the last training trial), mice were given a 1.5-min probe testing trial to test for short-retention memory. All holes on the maze were blocked, including the target hole. Each mouse was placed on the center of the maze to explore and visit the location where the escape box used to be. Three parameters were recorded during training and testing trials, including the primary distance which is the total distance traveled by mice to find the escape hole, the primary latency which is the time that an animal took to encounter the escape hole for the first time, and the search strategies which are defined as random (animals move randomly across the platform until the escape box is found), serial (animals travel through consecutive holes around the periphery of the maze until they find the escape box), and direct (animals navigate directly to the correct quadrant and escape box without crossing the maze center more than once and with less than two errors). The time spent in the target quadrant was recorded during probe testing trials. Data was analyzed using the EthoVision XT 17 system.

### Three-chamber sociability task

The three-chamber sociability task was used to measure a mouse’s social interaction. All animals were acclimated in the testing room at least 30 min before testing starts. The test was performed in a custom made matte black plastic box arena with three chambers and openings between the chambers. The two side chambers contain a plastic cylinder with holes allowing mice to interact with the object or mouse in the cylinder. During the habituation session, cylinders in the two side chambers were empty. An animal was placed in the middle chamber and allowed to explore the arena freely for 10 min. Then it is followed with 10 min testing session, during which a novel mouse was placed in the cylinder in one chamber and a novel object was placed in the cylinder in the other chamber. Novel mice were 4–5 months old wild type C57BL/6 mice of the same sex that the tested mice had never been exposed to previously. The time spent and number of entries into each chamber and with the cylinder indicating direct interaction were recorded and analyzed by the EthoVision XT 17 system.

### Immunohistochemistry

Mice were anesthetized with intraperitoneal injection of ketamine/xylazine solution (100 mg/kg/10 mg/kg, Butler Schein Animal Health), and then perfused intracardially with PBS followed by 4% paraformaldehyde (PFA) in PBS (pH 7.4). Brains were collected, post-fixed in 4% PFA in PBS for 48 h, and then paraffin embedded and processed by the Comparative Pathology Laboratory (CPL) in the Department of Pathology at the University of Iowa. Brains were sectioned through the midline at the thickness of 5 µm using a rotary microtome (Leica), mounted onto superfrost/plus slides (48311-703, VWR), and processed for immunohistochemistry (IHC). After deparaffinization, slides were antigen retrieved in 10 mM sodium citrate buffer (pH 6.0) in Decloaking chamber (Biocare Medical, Pacheco, CA) at 110°C for 15 min. Slides were treated with 3% H_2_O_2_ for 8 min to quench endogenous peroxidase activity and blocked for endogenous Avidin/Biotin (Dako, Santa Clara, CA). Then slides were blocked with 10% goat serum in PBST at room temperature for 1 h and incubated with primary antibody for MANF (1:200, ab67271) at room temperature for 2 h. After wash, slides were incubated with HRP-conjugated biotinylated secondary antibodies (1:200, BA-1000, Vector Laboratories Inc.) for 1 h and incubated with Avidin-biotin-peroxidase complex (ABC) (Vector Laboratories Inc.). The immunolabeling was visualized using DAB peroxidase (HRP) substrate kit (SK-4100, Vector Laboratories Inc.) and counterstained with hematoxylin for 1 min. Slides were imaged using Olympus BX81 microscope (Olympus).

### Immunoblotting

Protein was extracted from mice brains as described previously (Wang et al., 2015). Protein concentration was determined using the DC protein assay according to the manufacture’s instruction. For immunoblotting, 30 µg protein samples were separated on 12% polyacrylamide gels by electrophoresis and transferred to nitrocellulose membranes and blocked in 5% BSA/1xTBS/0.05% Tween-20 for 1 h at room temperature prior to incubation with primary antibodies at 4°C overnight. Subsequently, membranes were washed with TBST and incubated with secondary antibodies conjugated to horseradish peroxidase. Blots were developed using the Cytiva Amersham™ ECL™ Prime Western Blotting Detection Reagent on Chemi™Doc imaging system (Bio- Rad Laboratories, Hercules, CA). Band intensity was quantified using Image Lab software (Bio-Rad Laboratories).

### Statistical analysis

GraphPad Prism 9.0 (GraphPad software, La Jolla, CA) was used for all statistical analyses. Data were expressed as mean ± SEM. Two-way or three-way analysis of variance (ANOVA) followed by Tukey’s *post hoc* tests were used for behavioral tests and immunoblotting data. Fisher’s exact test was used for search strategies distribution in Barnes maze test. Results with *p* values of <0.05 were considered significant. G*Power (version 3.1.9.7) was used to determine the minimum sample size required. The statistical analyses used for each dataset are listed in Table 1. The *p* values from ANOVA and Tukey’s *post hoc* tests are also presented in the figures and figure legends.

**TABLE 1:** Statistical analyses.

Dataset	Type of analysis	Results
Figure 2B	Two-way ANOVA followed by Tukey’s *post hoc* test	Genotype F (1, 8) = 43.94, *p* = 0.0002; EtOH F (1, 8) = 0.1669, *p* = 0.6936; Interaction F (1, 8) = 0.0005205, *p* = 0.9824
Figure 2C	Two-way ANOVA followed by Tukey’s *post hoc* test	Genotype F (1, 8) = 165.9, *p* < 0.0001; EtOH F (1, 8) = 0.2833, *p* = 0.6090; Interaction F (1, 8) = 4.051, *p* = 0.0790
Figure 2D	Two-way ANOVA followed by Tukey’s *post hoc* test	Genotype F (1, 69) = 29.65, *p* < 0.0001; Sex F (1, 69) = 187.6, *p* < 0.0001; Interaction F (1, 69) = 0.7417, *p* = 0.3921
Figure 2E	Three-way ANOVA followed by Tukey’s *post hoc* test	Days F (12, 411) = 45.10, *p* < 0.0001; Genotype F (1, 411) = 0.3624, *p* = 0.5475; EtOH F (1, 411) = 124.9, *p* < 0.0001; Days × Genotype F (12, 411) = 4.772, *p* < 0.0001; Days × EtOH F (12, 411) = 2.672, *p* = 0.0018; Genotype × EtOH F (1, 411) = 55.22, *p* < 0.0001; Days × Genotype × EtOH F (12, 411) = 1.565, *p* = 0.0990
Figure 2F	Three-way ANOVA followed by Tukey’s *post hoc* test	Days F (11, 375) = 61.59, *p* < 0.0001; Genotype F (1, 375) = 0.02674, *p* = 0.8702; EtOH F (1, 375) = 557.2, *p* < 0.0001; Days × Genotype F (11, 375) = 1.490, *p* = 0.1327; Days × EtOH F (11, 375) = 10.46, *p* < 0.0001; Genotype × EtOH F (1, 375) = 12.39, *p* = 0.0005; Days × Genotype × EtOH F (11, 375) = 2.617, *p* = 0.0032
Figure 2G	Two-way ANOVA followed by Tukey’s *post hoc* test	Genotype F (1, 31) = 1.926, *p* = 0.1750; EtOH F (1, 31) = 19.42, *p* = 0.0001; Interaction F (1, 31) = 8.155, *p* = 0.0076
Figure 2H	Two-way ANOVA followed by Tukey’s *post hoc* test	Genotype F (1, 30) = 0.02139, *p* = 0.8847; EtOH F (1, 30) = 39.25, *p* < 0.0001; Interaction F (1, 30) = 0.4985, *p* = 0.4856
Figure 2I	Two-way ANOVA followed by Tukey’s *post hoc* test	Genotype F (1, 12) = 30.82, *p* = 0.0001; EtOH F (1, 12) = 0.2330, *p* = 0.6380; Interaction F (1, 12) = 2.097,*p* = 0.1732
Figure 2J	Two-way ANOVA followed by Tukey’s *post hoc* test	Genotype F (1, 11) = 0.6732, *p* = 0.4294; EtOH F (1, 11) = 0.3590, *p* = 0.5612; Interaction F (1, 11) = 0.1886, *p* = 0.6725
Figure 3A	Two-way ANOVA followed by Tukey’s *post hoc* test	12 mm: Genotype F (1, 31) = 2.910, *p* = 0.0980; EtOH F (1, 31) = 0.1390, *p* = 0.7118; Interaction F (1, 31) = 0.5054, *p* = 0.4825. 6 mm: Genotype F (1, 31) = 1.657, *p* = 0.2075; EtOH F (1, 31) = 0.01045, *p* = 0.9192; Interaction F (1, 31) = 0.8571, *p* = 0.3617
Figure 3B	Two-way ANOVA followed by Tukey’s *post hoc* test	12 mm: Genotype F (1, 29) = 0.6537, *p* = 0.4254; EtOH F (1, 29) = 1.672, *p* = 0.2062; Interaction F (1, 29) = 0.3010, *p* = 0.5874. 6 mm: Genotype F (1, 29) = 0.1020, *p* = 0.7518; EtOH F (1, 29) = 0.03614, *p* = 0.8505; Interaction F (1, 29) = 0.1363, *p* = 0.7147
Figure 3C	Two-way ANOVA followed by Tukey’s *post hoc* test	Genotype F (1, 31) = 12.22, *p* = 0.0014; EtOH F (1, 31) = 3.194, *p* = 0.0837; Interaction F (1, 31) = 0.01704, *p* = 0.8970
Figure 3D	Two-way ANOVA followed by Tukey’s *post hoc* test	Genotype F (1, 29) = 0.02052, *p* = 0.8871; EtOH F (1, 29) = 1.439, *p* = 0.2400; Interaction F (1, 29) = 0.4133, *p* = 0.5253
Figure 4A	Two-way ANOVA followed by Tukey’s *post hoc* test	Day 1: Genotype F (1, 31) = 8.850, *p* = 0.0056; EtOH F (1, 31) = 0.04919, *p* = 0.8259; Interaction F (1, 31) = 0.002640, *p* = 0.9594. Day 2: Genotype F (1, 31) = 11.20, *p* = 0.0022; EtOH F (1, 31) = 2.086, *p* = 0.1587; Interaction F (1, 31) = 0.07601, *p* = 0.7846
Figure 4B	Two-way ANOVA followed by Tukey’s *post hoc* test	Day 1: Genotype F (1, 29) = 1.783, *p* = 0.1921; EtOH F (1, 29) = 1.959, *p* = 0.1723; Interaction F (1, 29) = 1.099, *p* = 0.3032. Day 2: Genotype F (1, 29) = 2.119, *p* = 0.1562; EtOH F (1, 29) = 0.4067, *p* = 0.5287; Interaction F (1, 29) = 1.655, *p* = 0.2084
Figure 4C	Two-way ANOVA followed by Tukey’s *post hoc* test	Day 1: Genotype F (1, 31) = 3.378, *p* = 0.0757; EtOH F (1, 31) = 10.44, *p* = 0.0029; Interaction F (1, 31) = 0.08406, *p* = 0.7738. Day 2: Genotype F (1, 31) = 0.2457, *p* = 0.6236; EtOH F (1, 31) = 0.6549, *p* = 0.4245; Interaction F (1, 31) = 0.3322, *p* = 0.5685
Figure 4D	Two-way ANOVA followed by Tukey’s *post hoc* test	Day 1: Genotype F (1, 29) = 1.018, *p* = 0.3214; EtOH F (1, 29) = 0.7962, *p* = 0.3796; Interaction F (1, 29) = 0.09086, *p* = 0.7652. Day 2: Genotype F (1, 29) = 0.6773, *p* = 0.4173; EtOH F (1, 29) = 0.3265, *p* = 0.5721; Interaction F (1, 29) = 0.5743, *p* = 0.4547
Figure 4E	Two-way ANOVA followed by Tukey’s *post hoc* test	Female: Genotype F (1, 31) = 3.054, *p* = 0.0904; EtOH F (1, 31) = 0.1367, *p* = 0.7141; Interaction F (1, 31) = 0.06009, *p* = 0.8080. Male: Genotype F (1, 29) = 0.3267, *p* = 0.5720; EtOH F (1, 29) = 0.1163, *p* = 0.7355; Interaction F (1, 29) = 0.1329, *p* = 0.7181
Figure 4F	Two-way ANOVA followed by Tukey’s *post hoc* test	Female: Genotype F (1, 31) = 0.04051, *p* = 0.8418; EtOH F (1, 31) = 0.7014, *p* = 0.4087; Interaction F (1, 31) = 0.4508, *p* = 0.5070. Male: Genotype F (1, 29) = 0.3619, *p* = 0.5521; EtOH F (1, 29) = 0.7862, *p* = 0.3825; Interaction F (1, 29) = 2.021, *p* = 0.1658
Figure 5A	Three-way ANOVA followed by Tukey’s *post hoc* test	Days F (3, 112) = 35.31, *p* < 0.0001; Genotype F (1, 112) = 15.15, *p* = 0.0002; EtOH F (1, 112) = 0.1362, *p* = 0.7127; Days × Genotype F (3, 112) = 1.548, *p* = 0.2061; Days × EtOH F (3, 112) = 1.048, *p* = 0.3741; Genotype × EtOH F (1, 112) = 3.039, *p* = 0.0840; Days × Genotype × EtOH F (3, 112) = 0.2675, *p* = 0.8487
Figure 5B	Three-way ANOVA followed by Tukey’s *post hoc* test	Days F (3, 108) = 40.95, *p* < 0.0001; Genotype F (1, 108) = 0.06038, *p* = 0.8064; EtOH F (1, 108) = 6.394, *p* = 0.0129; Days × Genotyp F (3, 108) = 0.4196, *p* = 0.7393; Days × EtOH F (3, 108) = 1.777, *p* = 0.1559; Genotype × EtOH F (1, 108) = 3.231, *p* = 0.0750; Days × Genotype × EtOH F (3, 108) = 0.2918, *p* = 0.8313
Figure 5C	Three-way ANOVA followed by Tukey’s *post hoc* test	Days F (3, 112) = 18.32, *p* < 0.0001; Genotype F (1, 112) = 13.50, *p* = 0.0004; EtOH F (1, 112) = 0.6502, *p* = 0.4217; Days × Genotype F (3, 112) = 1.112, *p* = 0.3475; Days × EtOH F (3, 112) = 0.6379, *p* = 0.5921; Genotype × EtOH F (1, 112) = 0.1176, *p* = 0.7322; Days × Genotype × EtOH F (3, 112) = 0.2301, *p* = 0.8753
Figure 5D	Three-way ANOVA followed by Tukey’s *post hoc* test	Days F (3, 108) = 19.29, *p* < 0.0001; Genotype F (1, 108) = 0.2402, *p* = 0.6250; EtOH F (1, 108) = 5.426, *p* = 0.0217; Days × Genotype F (3, 108) = 0.8728, *p* = 0.4576; Days × EtOH F (3, 108) = 1.325, *p* = 0.2702; Genotype × EtOH F (1, 108) = 0.2602, *p* = 0.6110; Days × Genotype × EtOH F (3, 108) = 0.3981, *p* = 0.7547
Figure 5E	Two-way ANOVA followed by Tu s *post hoc* test	Genotype F (1, 28) = 0.9569, *p* = 0.3363; EtOH F (1, 28) = 0.2613, *p* = 0.6132; Interaction F (1, 28) = 1.635, *p* = 0.2115
Figure 5F	Two-way ANOVA followed by Tukey’s *post hoc* test	Genotype F (1, 27) = 1.839, *p* = 0.1863; EtOH F (1, 27) = 9.209, *p* = 0.0053; Interaction F (1, 27) = 1.515, *p* = 0.2291
Figure 5G	Two-way ANOVA followed by Tukey’s *post hoc* test	Genotype F (1, 28) = 1.479, *p* = 0.2341; EtOH F (1, 28) = 0.2663, *p* = 0.6099; Interaction F (1, 28) = 3.082, *p* = 0.0901
Figure 5H	Two-way ANOVA followed by Tukey’s *post hoc* test	Genotype F (1, 27) = 0.09571, *p* = 0.7594; EtOH F (1, 27) = 7.196, *p* = 0.0123; Interaction F (1, 27) = 0.1300, *p* = 0.7212
Figure 5I	Two-way ANOVA followed by Tukey’s *post hoc* test	Genotype F (1, 28) = 5.915, *p* = 0.0217; EtOH F (1, 28) = 2.687, *p* = 0.1123; Interaction F (1, 28) = 0.04038, *p* = 0.8422
Figure 5J	Two-way ANOVA followed by Tukey’s *post hoc* test	Genotyp F (1, 27) = 3.462, *p* = 0.0737; EtOH F (1, 27) = 12.45, *p* = 0.0015; Interaction F (1, 27) = 2.172, *p* = 0.1521
Figure 6A	Fisher’s exact test	Day 1 *p* = 0.8247; Day 2 *p* = 0.9608; Day 3 *p* = 0.2203; Day 4 *p* = 0.9314
Figure 6B	Fisher’s exact test	*p* = 0.6361
Figure 6C	Fisher’s exact test	Day 1 *p* = 0.1100; Day 2 *p* = 0.0247; Day 3 *p* = 0.0619; Day 4 *p* = 0.5593
Figure 6D	Fisher’s exact test	*p* = 0.2286
Figure 7A	Two-way ANOVA followed by Tukey’s *post hoc* test	Genotype F (1, 31) = 0.7541, *p* = 0.3919; EtOH F (1, 31) = 1.004, *p* = 0.3240; Interaction F (1, 31) = 0.1098, *p* = 0.7426
Figure 7B	Two-way ANOVA followed by Tukey’s *post hoc* test	Genotype F (1, 29) = 0.03993, *p* = 0.8430; EtOH F (1, 29) = 0.1442, *p* = 0.7069; Interaction F (1, 29) = 0.5018, *p* = 0.4844
Figure 7C	Three-way ANOVA followed by Tukey’s *post hoc* test	Chamber F (2, 93) = 185.1, *p* < 0.0001; Genotype F (1, 93) = 0.0001112, *p* = 0.9916; EtOH F (1, 93) = 3.250e-005, *p* = 0.9955; Chamber × Genotype F (2, 93) = 1.432, *p* = 0.2441; Chamber × EtOH F (2, 93) = 0.8018, *p* = 0.4516; Genotype × EtOH F (1, 93) = 0.0001531, *p* = 0.9902; Chamber × Genotype × EtOH F (2, 93) = 2.177, *p* = 0.1191
Figure 7D	Three-way ANOVA followed by Tukey’s *post hoc* test	Chamber F (2, 87) = 111.2, *p* < 0.0001; Genotype F (1, 87) = 2.043e-006, *p* = 0.9989; EtOH F (1, 87) = 3.783e-007, *p* = 0.9995; Chamber × Genotype F (2, 87) = 2.436, *p* = 0.0935; Chamber × EtOH F (2, 87) = 0.5794, *p* = 0.5624; Genotype × EtOH F (1, 87) = 7.083e-007, *p* = 0.9993; Chamber × Genotype × EtOH F (1, 87) = 7.083e-007, *p* = 0.9993
Figure 7E	Three-way ANOVA followed by Tukey’s *post hoc* test	Chamber F (1, 62) = 125.2, *p* < 0.0001; Genotype F (1, 62) = 10.36, *p* = 0.0021; EtOH F (1, 62) = 0.05816, *p* = 0.8102; Chamber × Genotype F (1, 62) = 5.839, *p* = 0.0186; Chamber × EtOH F (1, 62) = 0.3564, *p* = 0.5527; Genotype × EtOH F (1, 62) = 0.05193, *p* = 0.8205; Chamber × Genotype × EtOH F (1, 62) = 0.5256, *p* = 0.4712
Figure 7F	Three-way ANOVA followed by Tukey’s *post hoc* test	Chamber F (1, 58) = 148.4, *p* < 0.0001; Genotype F (1, 58) = 11.76, *p* = 0.0011; EtOH F (1, 58) = 1.469, *p* = 0.2305; Chamber × Genotype F (1, 58) = 6.695, *p* = 0.0122; Chamber × EtOH F (1, 58) = 2.961, *p* = 0.0906; Genotype × EtOH F (1, 58) = 0.8447, *p* = 0.3619; Chamber × Genotype × EtOH F (1, 58) = 0.3260, *p* = 0.5702
Figure 7G	Three-way ANOVA followed by Tukey’s *post hoc* test	Chamber F (1, 62) = 102.8, *p* < 0.0001; Genotype F (1, 62) = 5.183, *p* = 0.0263; EtOH F (1, 62) = 0.8930, *p* = 0.3483; Chamber × Genotype F (1, 62) = 2.819, *p* = 0.0982; Chamber × EtOH F (1, 62) = 0.003396, *p* = 0.9537; Genotype × EtOH F (1, 62) = 0.2230, *p* = 0.6385; Chamber × Genotype × EtOH F (1, 62) = 2.637, *p* = 0.1094
Figure 7H	Three-way ANOVA followed by Tukey’s *post hoc* test	Chamber F (1, 58) = 84.95, *p* < 0.0001; Genotype F (1, 58) = 5.943, *p* = 0.0179; EtOH F (1, 58) = 0.5688, *p* = 0.4538; Chamber × Genotype F (1, 58) = 3.489, *p* = 0.0668; Chamber × EtOH F (1, 58) = 0.3052, *p* = 0.5827; Genotype × EtOH F (1, 58) = 0.01969, *p* = 0.8889; Chamber × Genotype × EtOH F (1, 58) = 0.01300, *p* = 0.9096
Figure 8B	Two-way ANOVA followed by Tukey’s *post hoc* test	GRP78: Genotype F (1, 8) = 24.92, *p* = 0.0011; EtOH F (1, 8) = 15.91, *p* = 0.0040; Interaction F (1, 8) = 24.89, *p* = 0.0011. GRP94: Genotype F (1, 8) = 39.00, *p* = 0.0002; EtOH F (1, 8) = 6.783, *p* = 0.0314; Interaction F (1, 8) = 3.825, *p* = 0.0862. p-IRE1α: Genotype F (1, 8) = 24.12, *p* = 0.0012; EtOH F (1, 8) = 24.12, *p* = 0.0012; Interaction F (1, 8) = 0.008512, *p* = 0.9288. XBP1s: Genotype F (1, 8) = 56.73, *p* < 0.0001; EtOH F (1, 8) = 2.589, *p* = 0.1463; Interaction F (1, 8) = 8.079, *p* = 0.0217. p-PERK: Genotype F (1, 8) = 3.497, *p* = 0.0984; EtOH F (1, 8) = 3.191, *p* = 0.1119; Interaction F (1, 8) = 0.4602, *p* = 0.5167
Figure 8D	Two-way ANOVA followed by Tukey’s *post hoc* test	GRP78: Genotype F (1, 8) = 38.57, *p* = 0.0003; EtOH F (1, 8) = 13.25, *p* = 0.0066; Interaction F (1, 8) = 1.712, *p* = 0.2271. GRP94: Genotype F (1, 8) = 59.46, *p* < 0.0001; EtOH F (1, 8) = 4.051, *p* = 0.0789; Interaction F (1, 8) = 16.42, *p* = 0.0037. p-IRE1α: Genotype F (1, 8) = 2.447, *p* = 0.1564; EtOH F (1, 8) = 0.6831, *p* = 0.4325; Interaction F (1, 8) = 0.1737, *p* = 0.6878. XBP1s: Genotype F (1, 8) = 0.07082, *p* = 0.7969; EtOH F (1, 8) = 0.05923, *p* = 0.8138; Interaction F (1, 8) = 3.354, *p* = 0.1044. p-PERK: Genotype F (1, 8) = 15.78, *p* = 0.0041; EtOH F (1, 8) = 0.3673, *p* = 0.5613; Interaction F (1, 8) = 2.028, *p* = 0.1923
Figure 8F	Two-way ANOVA followed by Tukey’s *post hoc* test	GRP78: Genotype F (1, 8) = 0.4315, *p* = 0.5297; EtOH F (1, 8) = 0.1173, *p* = 0.7408; Interaction F (1, 8) = 2.208, *p* = 0.1756. GRP94: Genotype F (1, 8) = 4.692, *p* = 0.0622; EtOH F (1, 8) = 4.500, *p* = 0.0667; Interaction F (1, 8) = 7.212, *p* = 0.0277. p-IRE1α: Genotype F (1, 8) = 0.3840, *p* = 0.5527; EtOH F (1, 8) = 0.0002587, *p* = 0.9876; Interaction F (1, 8) = 0.003785, *p* = 0.9525. XBP1s: Genotype F (1, 8) = 0.6569, *p* = 0.4411; EtOH F (1, 8) = 3.302, *p* = 0.1067; Interaction F (1, 8) = 3.613, *p* = 0.0938. p-PERK: Genotype F (1, 8) = 3.613, *p* = 0.0938; EtOH F (1, 8) = 0.2887, *p* = 0.6056; Interaction F (1, 8) = 6.670, *p* = 0.0325
Figure 8H	Two-way ANOVA followed by Tukey’s *post hoc* test	GRP78: Genotype F (1, 8) = 18.54, *p* = 0.0026; EtOH F (1, 8) = 9.296, *p* = 0.0158; Interaction F (1, 8) = 10.84, *p* = 0.0110. GRP94: Genotype F (1, 8) = 8.050, *p* = 0.0219; EtOH F (1, 8) = 13.87, *p* = 0.0058; Interaction F (1, 8) = 20.16, *p* = 0.0020. p-IRE1α: Genotype F (1, 8) = 3.268, *p* = 0.1082; EtOH F (1, 8) = 0.2024, *p* = 0.6648; Interaction F (1, 8) = 3.769, *p* = 0.0882. XBP1s: Genotype F (1, 8) = 0.03888, *p* = 0.8486; EtOH F (1, 8) = 1.068, *p* = 0.3317; Interaction F (1, 8) = 3.813, *p* = 0.0866. p-PERK: Genotype F (1, 8) = 23.98, *p* = 0.0012; EtOH F (1, 8) = 0.2747, *p* = 0.6144; Interaction F (1, 8) = 2.483, *p* = 0.1537
Figure 9B	Two-way ANOVA followed by Tukey’s *post hoc* test	IL6: Genotype F (1, 8) = 14.69, *p* = 0.0050; EtOH F (1, 8) = 6.782, *p* = 0.0314; Interaction F (1, 8) = 6.782, *p* = 0.0314. IBA1: Genotype F (1, 8) = 5.134, *p* = 0.0532; EtOH F (1, 8) = 3.414, *p* = 0.1018; Interaction F (1, 8) = 4.102, *p* = 0.0774. MCP1: Genotype F (1, 8) = 63.86, *p* < 0.0001; EtOH F (1, 8) = 63.86, *p* < 0.0001; Interaction F (1, 8) = 8.164, *p* = 0.0212. CCR2: Genotype F (1, 8) = 1.427, *p* = 0.2664; EtOH F (1, 8) = 1.427, *p* = 0.2664; Interaction F (1, 8) = 1.427, *p* = 0.2664
Figure 9D	Two-way ANOVA followed by Tukey’s *post hoc* test	IL6: Genotype F (1, 8) = 115.9, *p* < 0.0001; EtOH F (1, 8) = 10.51, *p* = 0.0118; Interaction F (1, 8) = 9.551, *p* = 0.0149. IBA1: Genotype F (1, 8) = 2.301, *p* = 0.1677; EtOH F (1, 8) = 0.004215, *p* = 0.9498; Interaction F (1, 8) = 2.937, *p* = 0.1249. MCP1: Genotype F (1, 8) = 13.81, *p* = 0.0059; EtOH F (1, 8) = 5.619, *p* = 0.0452; Interaction F (1, 8) = 6.570, *p* = 0.0335. CCR2: Genotype F (1, 8) = 11.31, *p* = 0.0099; EtOH F (1, 8) = 11.31, *p* = 0.0099; Interaction F (1, 8) = 2.643, *p* = 0.1427
Figure 9F	Two-way ANOVA followed by Tukey’s *post hoc* test	IL6: Genotype F (1, 8) = 4.323, *p* = 0.0712; EtOH F (1, 8) = 4.323, *p* = 0.0712; Interaction F (1, 8) = 0.002557, *p* = 0.9609. IBA1: Genotype F (1, 8) = 0.002557, *p* = 0.9609; EtOH F (1, 8) = 0.007818, *p* = 0.9317; Interaction F (1, 8) = 4.314, *p* = 0.0715. MCP1: Genotype F (1, 8) = 1.445e-005, *p* = 0.9971; EtOH F (1, 8) = 4.426, *p* = 0.0685; Interaction F (1, 8) = 0.1023, *p* = 0.7572. CCR2: Genotype F (1, 8) = 8.218, *p* = 0.0209; EtOH F (1, 8) = 0.01094, *p* = 0.9193; Interaction F (1, 8) = 0.001426, *p* = 0.9708
Figure 9H	Two-way ANOVA followed by Tukey’s *post hoc* test	IL6: Genotype F (1, 8) = 0.3189, *p* = 0.5877; EtOH F (1, 8) = 1.952, *p* = 0.1999; Interaction F (1, 8) = 2.247, *p* = 0.1723. IBA1: Genotype F (1, 8) = 35.54, *p* = 0.0003; EtOH F (1, 8) = 5.724, *p* = 0.0437; Interaction F (1, 8) = 6.199, *p* = 0.0375. MCP1: Genotype F (1, 8) = 6.199, *p* = 0.0375; EtOH F (1, 8) = 0.3670, *p* = 0.5615; Interaction F (1, 8) = 0.01426, *p* = 0.9079. CCR2: Genotype F (1, 8) = 13.72, *p* = 0.0060; EtOH F (1, 8) = 16.31, *p* = 0.0037; Interaction F (1, 8) = 12.39, *p* = 0.0079
Figure 10B	Two-way ANOVA followed by Tukey’s *post hoc* test	Neuroplastin: Genotype F (1, 8) = 34.25, *p* = 0.0004; EtOH F (1, 8) = 2.609, *p* = 0.1449; Interaction F (1, 8) = 3.332, *p* = 0.1054. PDIA1: Genotype F (1, 8) = 19.95, *p* = 0.0021; EtOH F (1, 8) = 4.407, *p* = 0.0690; Interaction F (1, 8) = 6.333, *p* = 0.0360. PDIA6: Genotype F (1, 8) = 25.23, *p* = 0.0010; EtOH F (1, 8) = 15.17, *p* = 0.0046; Interaction F (1, 8) = 3.476, *p* = 0.0993
Figure 10D	Two-way ANOVA followed by Tukey’s *post hoc* test	Neuroplastin: Genotype F (1, 8) = 150.7, *p* < 0.0001; EtOH F (1, 8) = 96.49, *p* < 0.0001; Interaction F (1, 8) = 9.326, *p* = 0.0157. PDIA1: Genotype F (1, 8) = 61.02, *p* < 0.0001; EtOH F (1, 8) = 26.95, *p* = 0.0008; Interaction F (1, 8) = 47.79, *p* = 0.0001. PDIA6: Genotype F (1, 8) = 212.1, *p* < 0.0001; EtOH F (1, 8) = 65.39, *p* < 0.0001; Interaction F (1, 8) = 35.17, *p* = 0.0003
Figure 10F	Two-way ANOVA followed by Tukey’s *post hoc* test	Neuroplastin: Genotype F (1, 8) = 3.247, *p* = 0.1092; EtOH F (1, 8) = 8.863, *p* = 0.0177; Interaction F (1, 8) = 3.844, *p* = 0.0856. PDIA1: Genotype F (1, 8) = 4.336, *p* = 0.0709; EtOH F (1, 8) = 0.0008919, *p* = 0.9769; Interaction F (1, 8) = 1.261, *p* = 0.2940. PDIA6: Genotype F (1, 8) = 5.684, *p* = 0.0443; EtOH F (1, 8) = 1.727, *p* = 0.2252; Interaction F (1, 8) = 1.373e-005, *p* = 0.9971
Figure 10H	Two-way ANOVA followed by Tukey’s *post hoc* test	Neuroplastin: Genotype F (1, 8) = 80.37, *p* < 0.0001; EtOH F (1, 8) = 14.50, *p* = 0.0052; Interaction F (1, 8) = 0.9941, *p* = 0.3479. PDIA1: Genotype F (1, 8) = 26.09, *p* = 0.0009; EtOH F (1, 8) = 0.03967, *p* = 0.8471; Interaction F (1, 8) = 3.087, *p* = 0.1170. PDIA6: Genotype F (1, 8) = 29.83, *p* = 0.0006; EtOH F (1, 8) = 22.84, *p* = 0.0014; Interaction F (1, 8) = 38.57, *p* = 0.0003

## Results

### Effects of alcohol exposure and neuronal MANF deficiency on body and brain weight

**FIGURE 2:**
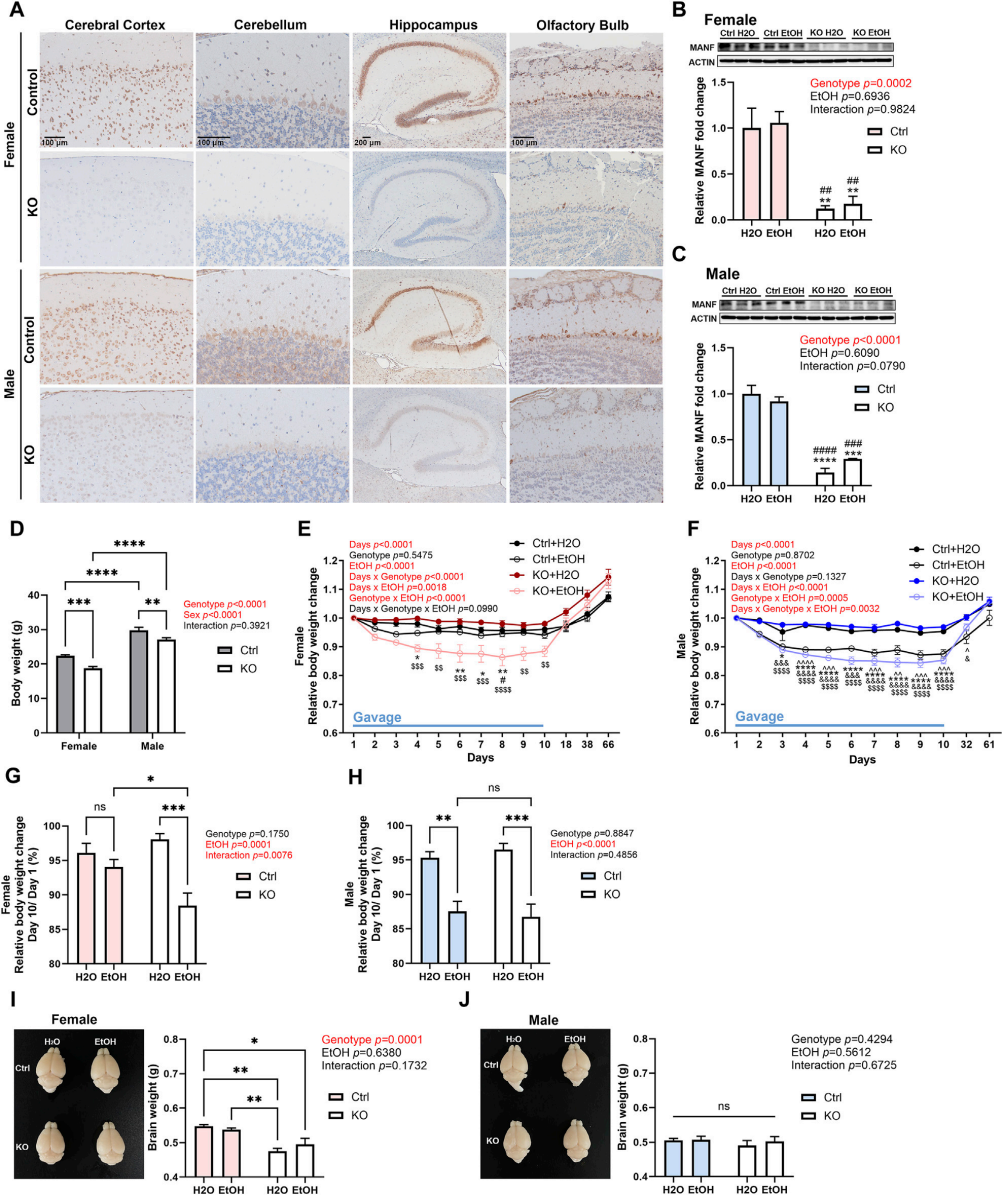
Effects of alcohol exposure and neuronal MANF deficiency on body and brain weight. **(A)** Representative images for immunohistochemical staining of MANF in control and MANF KO adult mice cerebral cortex, cerebellum, hippocampus, and olfactory bulb. **(B, C)** Representative immunoblots of MANF in the cerebral cortex of water- and alcohol-exposed control and MANF KO female **(B)** and male **(C)** mice. The data was expressed as mean ± SEM. n = 3 per group. Two-way ANOVA followed by Tukey’s *post hoc* test. **p < 0.01, ***p < 0.001, ****p < 0.0001 when compared with H_2_O treated control; ##*p* < 0.01, ###*p* < 0.001, ####*p* < 0.0001 when compared with ethanol treated control. **(D)** Body weight of control and MANF KO female and male. The data was expressed as mean ± SEM. n = 17–19 per group. Two-way ANOVA followed by Tukey’s *post hoc* test. **p < 0.01, ***p < 0.001, ****p < 0.0001. **(E, F)** Relative body weight change during gavage and behavior tests in female **(E)** and male **(F)** The data was expressed as mean ± SEM. n = 8–10 per group. Three-way ANOVA followed by Tukey’s *post hoc* test. ^*p* < 0.05, ^^*p* < 0.01, ^^^*p* < 0.001, ^^^^*p* < 0.0001 Ctrl + H_2_O v.s. Ctrl + EtOH; *p < 0.05, **p < 0.01, ****p < 0.0001 Ctrl + H_2_O v.s. KO + EtOH; #*p* < 0.05 Ctrl + EtOH v.s. KO + EtOH; &*p* < 0.05, &&&*p* < 0.001, &&&&*p* < 0.0001 KO + H_2_O v.s. Ctrl + EtOH; $$*p* < 0.01, $$$$*p* < 0.0001 KO + H_2_O v.s. KO + EtOH. **(G, H)** Relative body weight change on day 10 when compared to day 1 during gavage in female **(G)** and male **(H)** The data was expressed as mean ± SEM. n = 7–9 per group. Two-way ANOVA followed by Tukey’s *post hoc* test. *p < 0.05, **p < 0.01, ***p < 0.001. **(I, J)** Representative images of the brain and average brain weight in female **(I)** and male **(J)** The data was expressed as mean ± SEM. n = 3–4 per group. Two-way ANOVA followed by Tukey’s *post hoc* test. *p < 0.05; **p < 0.01; ns not significant.

Neuron-specific MANF KO mice were generated as described previously (Pakarinen et al., 2020; Wang et al., 2021). Immunohistochemistry (IHC) revealed that MANF protein was strongly expressed in the brain of control adult mice and was significantly reduced in both male and female MANF KO mice in all the brain regions analyzed, including the cerebral cortex, cerebellum, hippocampus, and olfactory bulb (Figure 2A). Similarly, immunoblot also confirmed the ablation of MANF protein in the cerebral cortex of MANF KO mice (Figures 2B,C). The initial body weight of all animals was recorded (Figure 2D). Notably, females had lower body weight than males, and MANF KO mice had lower body weight when compared to controls, which could be potentially due to the Nestin-Cre background (Declercq et al., 2015; Pakarinen et al., 2020). While water gavage had minimal effect on body weight, alcohol gavage resulted in a rapid body weight loss during the first 5 days. Then the rate of body weight reduction slowed down. Alcohol-treated groups remained to have lower body weight than water-treated groups by the end of day 10 for female KO, male control and KO (Figures 2E,F), but not for female control (Figure 2G). At the end of 10 days gavage, alcohol induced 5.942% ± 1.105% of weight loss in female controls, but significantly more in female MANF KO animals (11.567% ± 1.842%) (Figure 2G). Alcohol-associated weight loss was comparable between male controls (12.455% ± 1.454%) and male MANF KO (14.711% ± 1.233%) (Figure 2H). Body weight recovered to pregavage level 1 month after the last gavage and kept rising steadily (Figures 2E,F). Brain weight was not affected by alcohol treatment, but in females, the brain weight of KO mice was less than that of control mice (Figure 2I). In males, however, there was no difference in brain weight among groups (Figure 2J).

### Effects of alcohol exposure and neuronal MANF deficiency on motor functions

**FIGURE 3:**
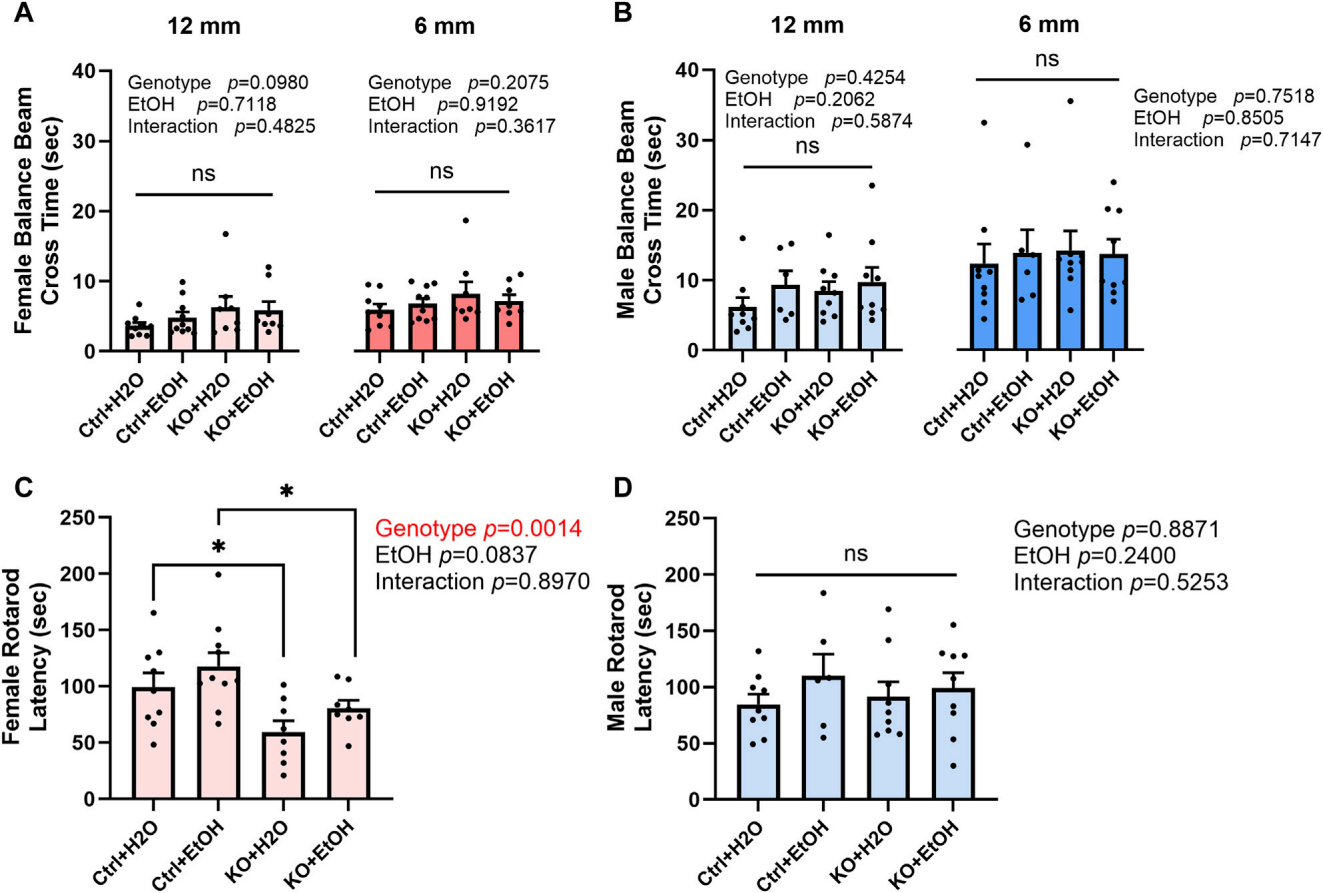
Effects of alcohol exposure and neuronal MANF deficiency on motor functions in balance beam and rotarod tests. **(A, B)** Results of balance beam tests in female **(A)** and male **(B)**. The time to cross the 12 mm and 6 mm beams in each treatment group was expressed as mean ± SEM. n = 6–10 per group. Two-way ANOVA followed by Tukey’s *post hoc* test. **(C, D)** The latency to fall from the accelerating rotarod in female **(C)** and male **(D)**. The data was expressed as mean ± SEM. n = 6–10 per group. Two-way ANOVA followed by Tukey’s *post hoc* test. *p < 0.05; ns not significant.

The impact of alcohol exposure and MANF deficiency on motor function was evaluated by the tests of balance beam, rotarod, and DigiGait. Results from balance beam test showed that neither alcohol exposure nor MANF deficiency affected the cross time (Figures 3A,B). Then animals were tested in rotarod tests. In females, the latency to fall was significantly affected by genotype but not alcohol. *Post hoc* analysis detected that MANF KO females fell significantly quicker than control females regardless of treatment (Figure 3C). No significant difference was observed in males among any groups (Figure 3D). It should be noted that the reduced body weight in MANF KO animals can potentially affect muscle strength and time that animals spend on the rotarod. As a result, the reduced latency to fall from the rotarod in female KO can be related to the body weight deficit due to Nestin-Cre background (Giusti et al., 2014).

To further examine if alcohol exposure or MANF deficiency had an impact on gait or walking patterns, animals were tested in DigiGait test. All animals were tested with three belt speeds: 10, 20, and 30 cm/s. As expected, fewer animals were able to complete the test with increased belt speed (data not shown). Both spatial and temporal parameters from the DigiGait test were analyzed. Spatial parameters include paw area, step angle, stance width, and stride length; temporal parameters include stride time, swing time, braking time, and propulsion time (Akula et al., 2020). No difference was observed in any of the parameters (data not shown).

These above results indicated that motor function was not affected by acute binge alcohol exposure nor MANF KO, with the exception that female MANF KO mice showed a reduced latency to fall in the rotarods test.

### Effects of alcohol exposure and neuronal MANF deficiency on anxiety-like behaviors

**FIGURE 4:**
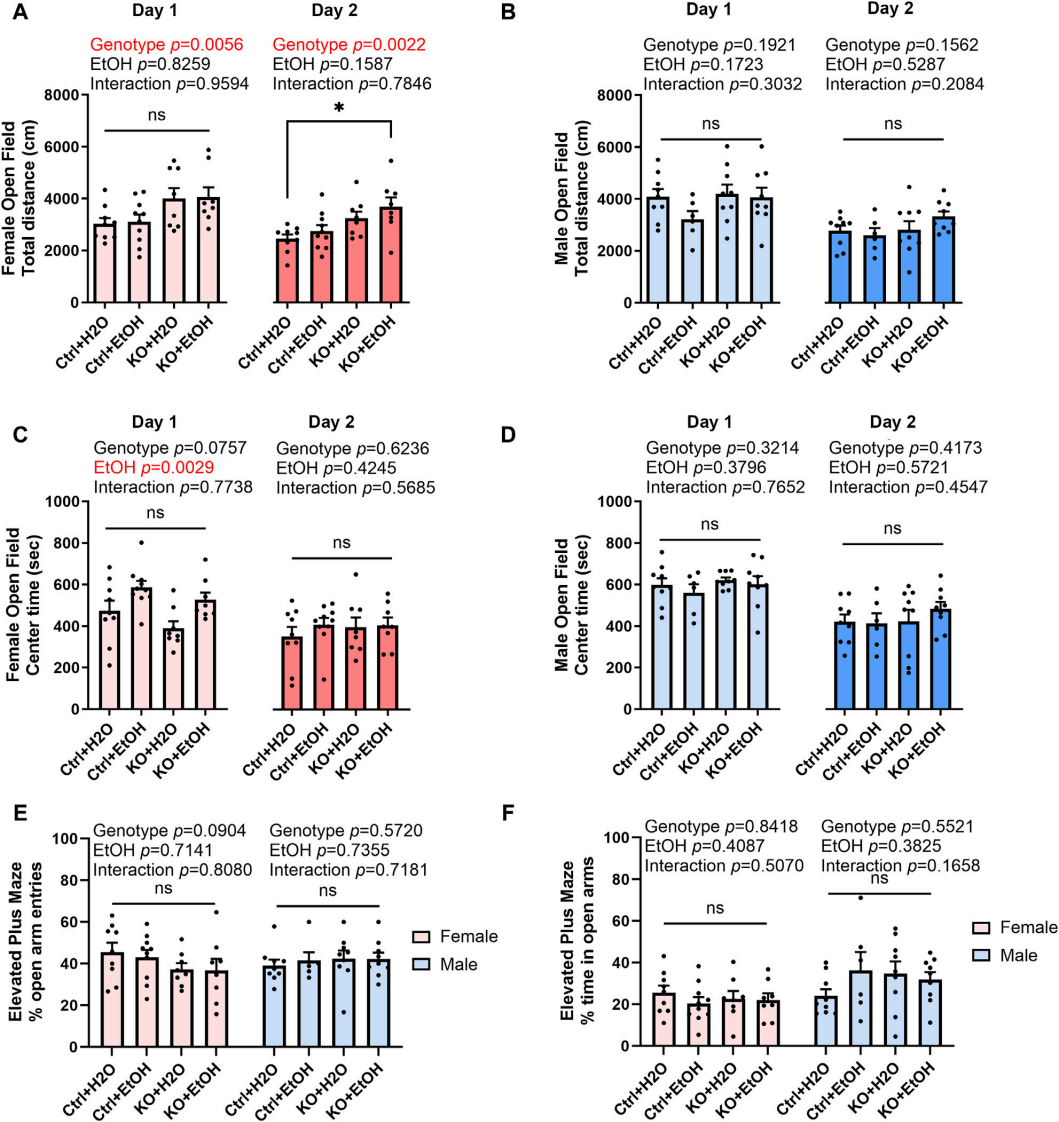
Effects of binge alcohol exposure and neuronal MANF deficiency on anxiety-like behaviors in open field and elevated plus maze tests. **(A, B)** The total distance (cm) traveled in the open filed arena at day 1 and day 2 in female **(A)** and male **(B)**. **(C, D)** The time (sec) spent in the center of the open field arena at day 1 and day 2 in female **(C)** and male **(D)**. **(E)** The percentage of open arm entries in female and male. **(F)** The percentage of time spent in the open arms in female and male. All data was expressed as mean ± SEM. n = 6–10 per group. Two-way ANOVA followed by Tukey’s *post hoc* test. *p < 0.05; ns not significant.

The comorbidity of anxiety with alcohol use disorder is very high (Schneier et al., 2010). To test if acute binge alcohol exposure can cause anxiety and whether it is affected by MANF deficiency, control and MANF KO animals were assessed in open field and elevated plus maze tests. The open field test was conducted for two consecutive days. The total distance traveled, and center time were recorded. In females, distance traveled was significantly affected by genotype for both day 1 and day 2. MANF KO females tended to travel longer total distance than control females. *Post hoc* analysis revealed that alcohol-exposed MANF KO female traveled significantly longer distance than water-treated control females on day 2 (Figure 4A). In males, total distance traveled was not affected by alcohol nor MANF deficiency (Figure 4B). These results indicated that female KO mice had a generally increased locomotor activity, but no effect was observed due to alcohol.

Reduced center time in open field test is associated with increased anxiety. In females, center time was significantly affected by alcohol treatment on day 1 (Figure 4C). Alcohol-exposed groups had a trend of increased center time when compared to water groups regardless of their genotypes. No main effects of genotype nor alcohol treatment were detected for day 2, probably because anxiety test results are heavily affected by the test repetition due to animal’s adaptation to the experimental environment (Võikar et al., 2004). In males, center time was not affected by alcohol nor MANF deficiency on both day 1 and day 2 (Figure 4D).

Animals were then tested in elevated plus maze test. The number of entries and time spent in open and closed arms were recorded. Decreased number of entries or time spent in the open arms is indicative of anxiety-like behavior. No difference was observed in any of the group for both female and male (Figures 4E,F). Data from center time in open field test and elevated plus maze test indicated that anxiety-like behavior was not observed in acute binge alcohol exposed control and neuronal MANF KO animals.

### Effects of alcohol exposure and neuronal MANF deficiency on learning and memory

**FIGURE 5:**
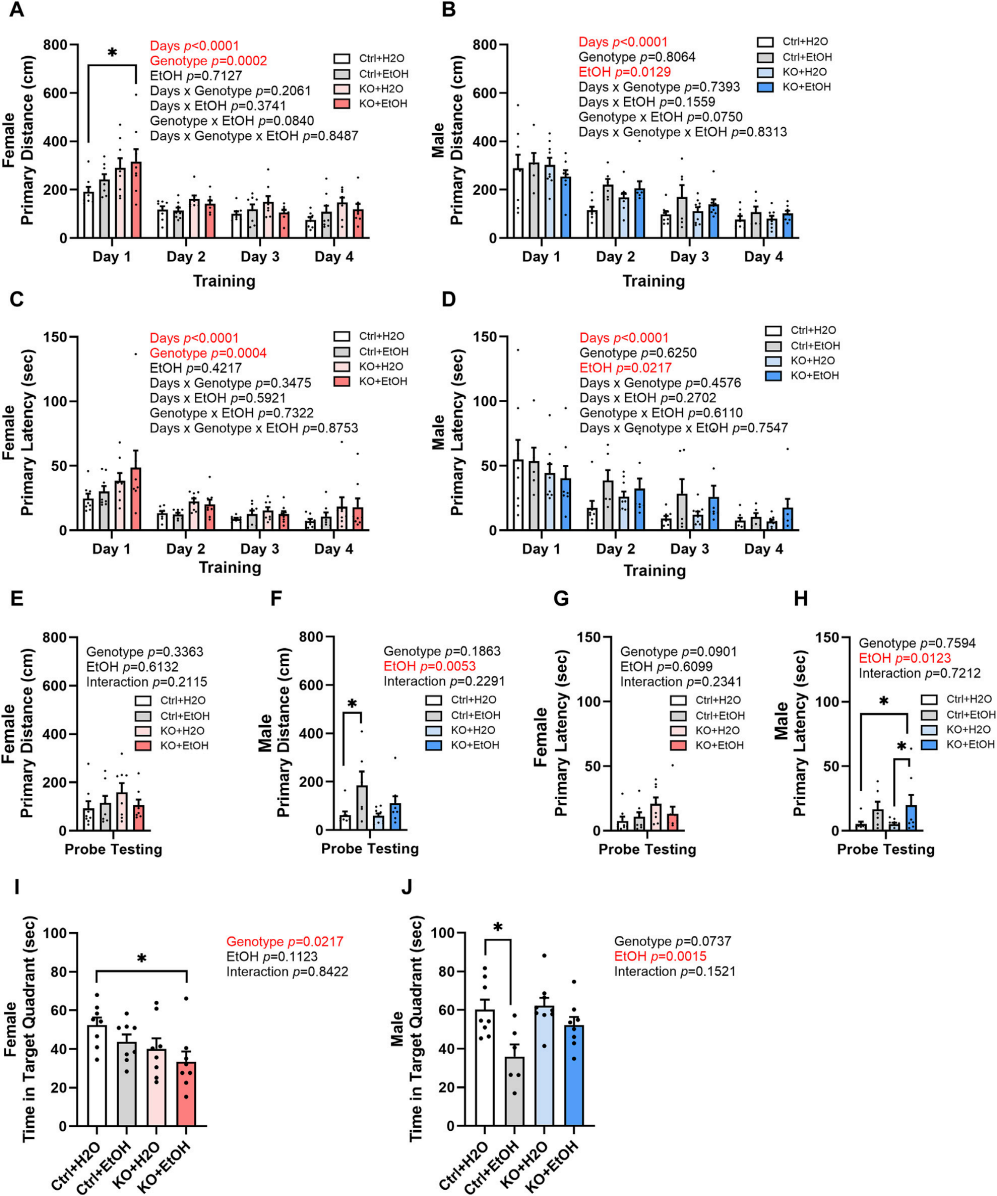
Effects of binge alcohol exposure and neuronal MANF deficiency on learning and memory in Barnes maze test. **(A, B)** Primary distance (cm) traveled by mice to find the escape hole during training days in female **(A)** and male **(B)**. **(C, D)** Primary latency (sec) to locate the escape hole during training days in female **(C)** and male **(D)**. **(E, F)** Primary distance (cm) traveled by mice to find the escape hole in probe testing day in female **(E)** and male **(F)**. **(G, H)** Primary latency (sec) to locate the escape hole in probe testing day in female **(G)** and male **(H)**. **(I, J)** Time (sec) spent in the target quadrant in probe testing day in female **(I)** and male **(J)**. All data were presented as mean ± SEM. n = 6–10 per group. **(A–D)**, three-way ANOVA followed by Tukey’s *post hoc* test. *p < 0.05; **(E–J)**, two-way ANOVA followed by Tukey’s *post hoc* test. *p < 0.05; **p < 0.01.

Binge alcohol drinking is associated with alterations in learning and memory and is a risk factor for the development of dementia (Obernier et al., 2002; Koch et al., 2019). Control and MANF KO animals were evaluated by Barnes maze test to determine their cognitive function in learning and memory. The primary distance traveled to find the escape hole and primary latency to escape were recorded. Over the four training days, a main effect of day was observed in primary distance and primary latency for both female and male (Figures 5A–D), indicating that all animals had the ability to learn. In female, primary distance and primary latency were also significantly affected by genotype. *Post hoc* analysis found that alcohol exposed MANF KO females had significantly increased primary distance than water treated controls on day 1 (Figure 5A). Results in males, however, were not affected by genotypes. Instead, it was significantly affected by alcohol. Individual group difference was not detected in *post hoc* analysis, but alcohol-exposed males tend to have longer primary distance and primary latency than water treated males on training day 2–4 (Figures 5B,D).

Animals were then tested in probe testing trials to assess their memory. In female, no difference in primary distance and latency was observed for probe testing trials (Figures 5E,G). However, a main effect of genotype was observed in the time spent in the target quadrant for females (Figure 5I). *Post hoc* analysis found that alcohol-exposed MANF KO females spent significantly shorter time than water-treated controls in the target quadrant (Figure 5I). Males again showed a main effect of alcohol in the probe testing trials regardless of their genotype. Alcohol-exposed male mice tend to travel longer distance and spent longer time to find the target hole and stayed shorter time in the target quadrant than the water-treated male mice (Figures 5F,H,J). *Post hoc* analysis revealed that alcohol-treated male control mice travelled significantly longer primary distance than water-treated control males (Figure 5F); they also showed shorter time spent in target quadrant when compared to water-treated control males (Figure 5J). These results suggest that there is a sex-dependent effect of alcohol exposure and MANF deficiency on learning and memory. Females were mainly affected by MANF deficiency, while males were affected by alcohol exposure.

**FIGURE 6:**
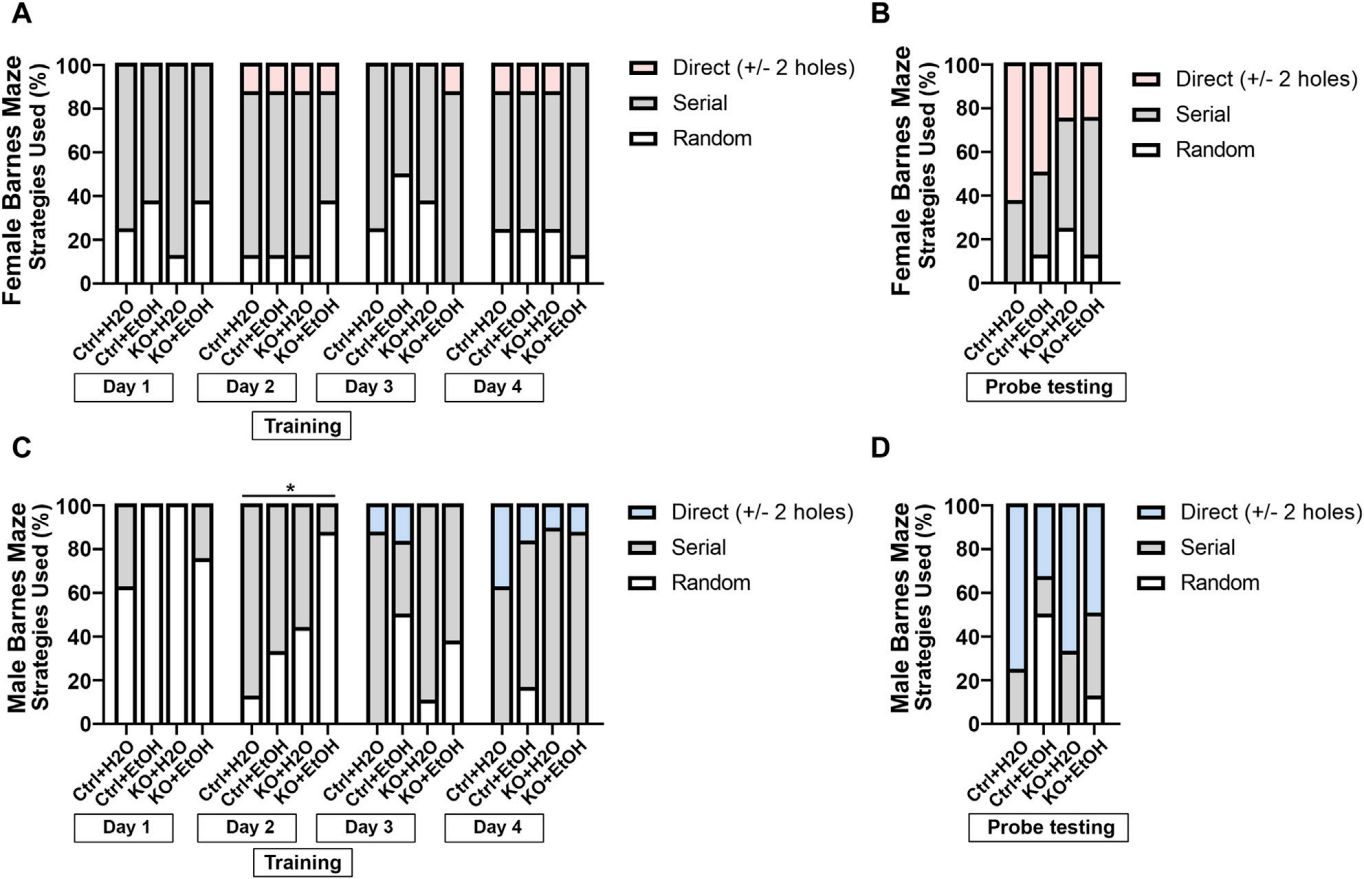
Searching strategies used by different experimental groups in Barnes maze test. **(A, B)** Percentage of each strategy used to find the escape hole during training **(A)** and probe testing period **(B)** in female. **(C, D)** Percentage of each strategy used to find the escape hole during training **(C)** and probe testing period **(D)** in male. The three defined search strategies are random (bottom), serial (middle), and direct (top). n = 6–10 per group. Fisher’s exact test. *p < 0.05.

We also examined animals’ search strategies in the Barnes maze test. It is characterized with three categories: 1. random, animals move randomly across the platform until the escape box is found; 2. serial, animals travel through consecutive holes around the periphery of the maze until they find the escape box; 3. direct, animals navigate directly to the correct quadrant and escape box without crossing the maze center more than once and with less than two errors. The frequency of the three strategies used during training and the probe testing days was analyzed to determine if alcohol exposure and MANF deficiency had an impact on the animals’ search strategy. As training days proceed, fewer animals used the random strategy, and more animals started to show direct search strategy (Figures 6A,C), which was indicative of the ability for learning. Fisher’s exact test indicated that the search strategy used by different groups of animals was significantly different for male animals on training day 2 (Figure 6C). On probe testing day, no statistic differences were detected, but we found a trend of difference for the use of search strategy between females and males. For females on probe testing day, we found an effect of MANF deficiency. While direct strategy was used by the majority of female control animals (Ctrl + H_2_O = 65%, Ctrl + EtOH = 50%), it was only used by a quarter of female MANF KO animals (KO + H_2_O = 25%, KO + EtOH = 25%) (Figure 6B). For males on probe testing day, we found an effect of alcohol treatment. More water-treated male animals used direct search strategy (Ctrl + H_2_O = 75%, KO + H_2_O = 67%) than that of alcohol-exposed male mice (Ctrl + EtOH = 33%, KO + EtOH = 50%) (Figure 6D). Together with the other Barnes maze test results, these results supported that although alcohol exposure and MANF deficiency did not significantly affect learning and memory, a sex-specific change of behavior was observed. In females learning and memory behavior was altered by MANF deficiency while in males it was affected by alcohol treatment.

### Effects of alcohol exposure and neuronal MANF deficiency on social behaviors

**FIGURE 7:**
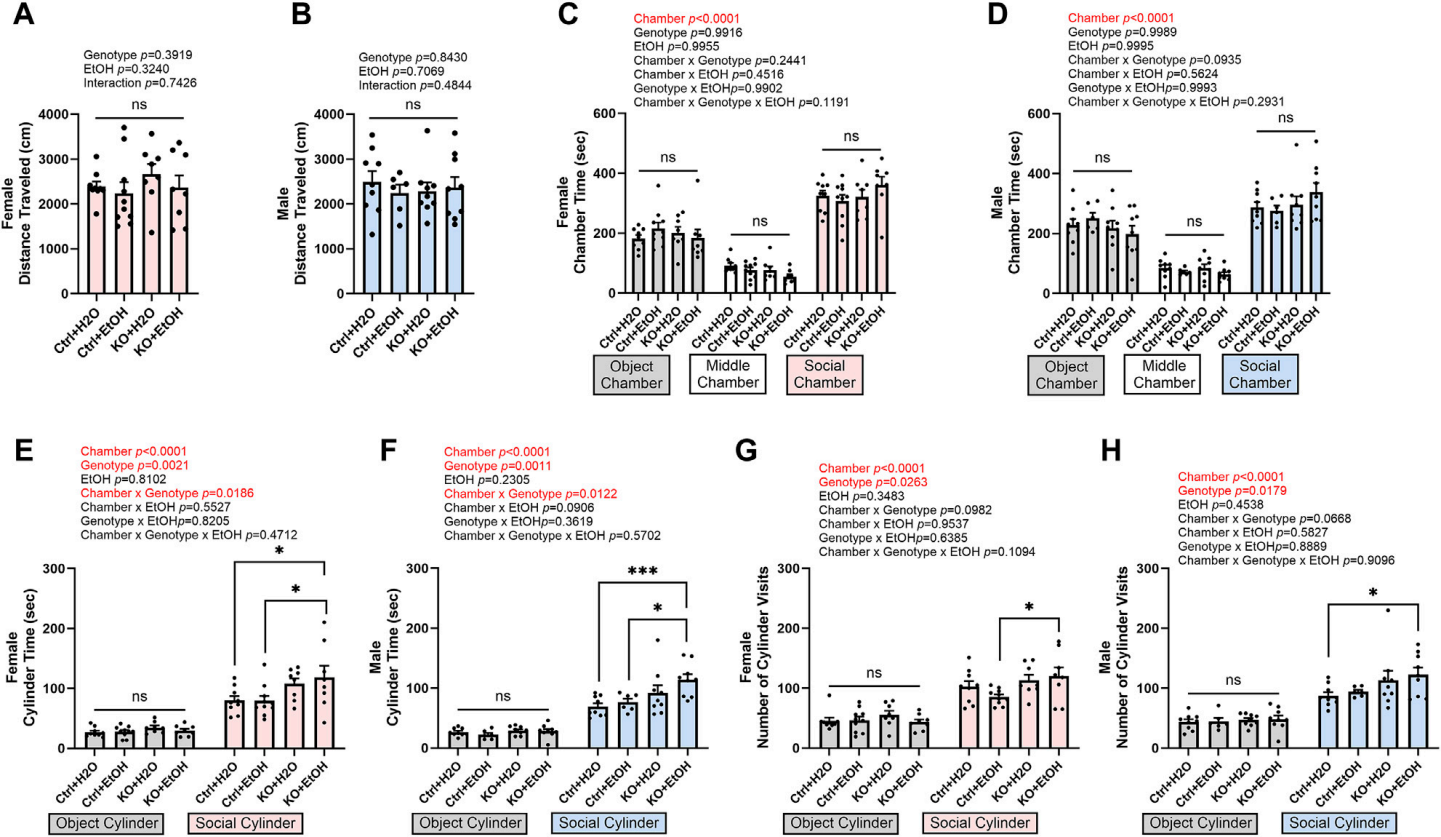
Effects of binge alcohol exposure and neuronal MANF deficiency on social behaviors in 3-chamber sociability test. **(A, B)** Total distance (cm) traveled in the test chambers in female **(A)** and male **(B)**. **(C, D)** Time (sec) spent in object, middle, and social chambers in female **(C)** and male **(D)**. **(E, F)** Time (sec) interacting with the object and social cylinder in female **(E)** and male **(F)**. **(G, H)** The number of object and social cylinder visits in female **(G)** and male **(H)**. Data were expressed as mean ± SEM. n = 6–10 per group. **(A, B)**, two-way ANOVA followed by Tukey’s *post hoc* test. **(C–H)**, three-way ANOVA followed by Tukey’s *post hoc* test. *p < 0.05; ***p < 0.001; ns not significant.

Binge alcohol drinking has been shown to increase sociability in both human and mice (Kirchner et al., 2006; Simon et al., 2023). To test whether social behavior was altered by acute alcohol binge exposure and MANF deficiency, mice were examined in the 3-chamber sociability test. The total distance traveled in the test chambers was not affected by alcohol exposure or MANF deficiency (Figures 7A,B), indicating the general activity and exploration was not affected. Both female and male mice showed a preference of the social chamber over the object chamber as they spent significantly longer time in the social chamber (Figures 7C,D). A main effect of genotype on sociability was observed for both male and female. Alcohol exposure did not alter the sociability in control animals. However, in both female and male MANF KO mice, alcohol exposure caused significantly longer interacting time and increased numbers of visit to the social cylinder when compared to the control animals (Figures 7E–H). This result suggested that MANF deficiency and alcohol binge exposure interacted to increase sociability in both female and male animals.

### Effects of alcohol exposure and neuronal MANF deficiency on neuronal ER homeostasis

**FIGURE 8:**
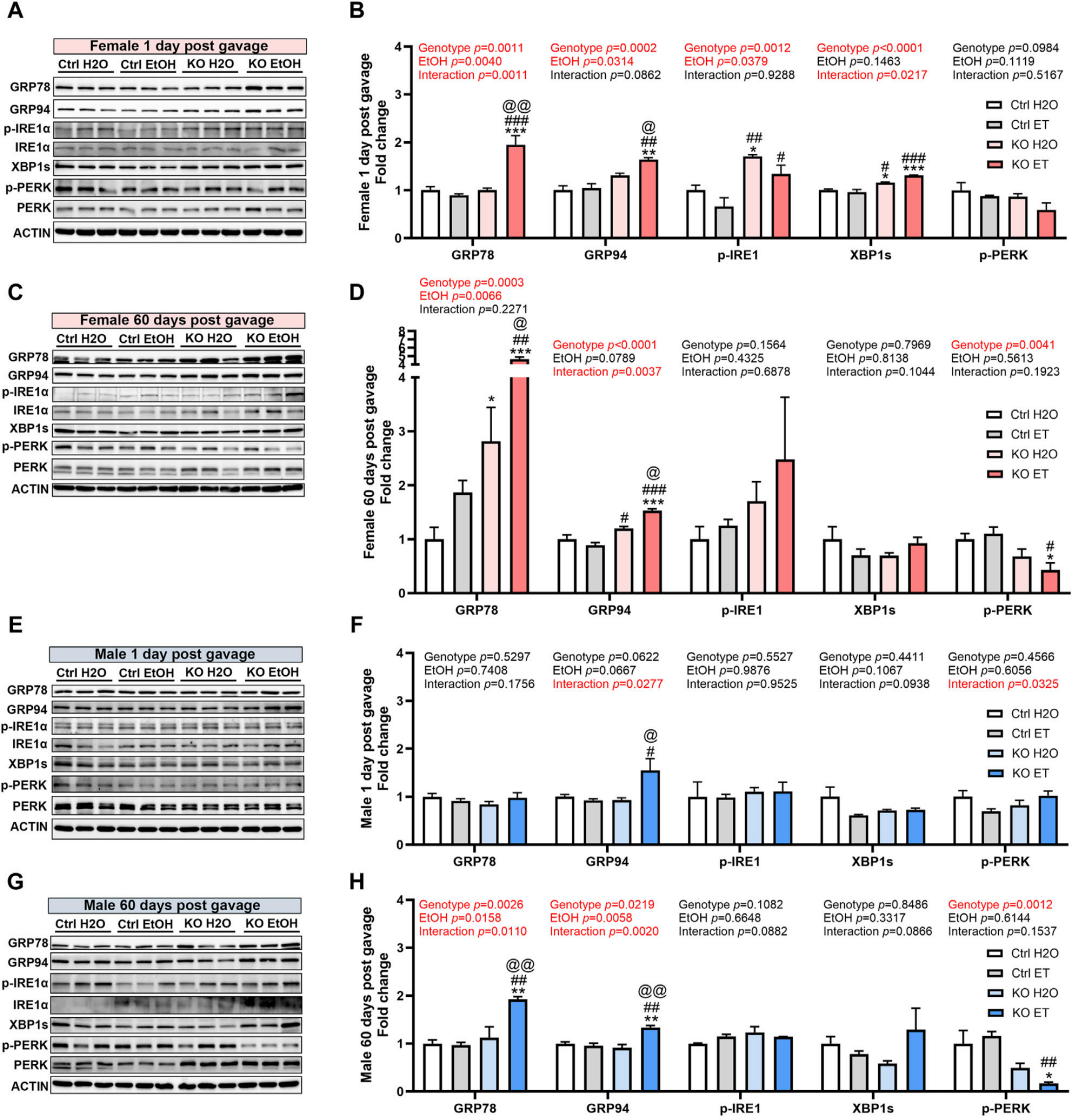
Effects of binge alcohol exposure and neuronal MANF deficiency on neuronal ER homeostasis. **(A–D)** Representative immunoblots **(A, C)** and quantification **(B, D)** of ER stress markers in female control and MANF KO cerebral cortex 1 day **(A, B)** or 60 days **(C–D)** post H_2_O or EtOH treatment. **(E, H)** Representative immunoblots **(E, G)** and quantification **(F, H)** of ER stress markers in male control and MANF KO cerebral cortex 1 day **(E, F)** or 60 days **(G, H)** post H_2_O or EtOH treatment. All data were expressed as mean ± SEM. n = 3 per group. Two-way ANOVA followed by Tukey’s *post hoc* test. *p < 0.05, **p < 0.01, ***p < 0.001 when compared to H_2_O treated control. #*p* < 0.05, ##*p* < 0.01, ###*p* < 0.001 when compared to EtOH treated control. @*p* < 0.05, @@*p* < 0.01 when compared to H_2_O treated KO.

We have previously shown that MANF deficiency exacerbated alcohol-induced neuronal apoptosis and ER stress in the developing brain (Wang et al., 2021) and binge-like alcohol exposure induce ER stress in the adult brain (Wang et al., 2018). To determine the effects of interaction of binge alcohol exposure and MANF deficiency on ER stress in the adult brain, we examined the expression of UPR proteins in the brain of control and MANF KO mice. Protein lysates were extracted from the cerebral cortex either 1 day or 60 days after the last gavage from two separate cohorts of animals. The expression of ER molecular chaperones GRP78 and GRP94, and other UPR proteins were examined. While UPR was not activated by alcohol in control animals, the expression of several UPR proteins were altered in alcohol exposed MANF KO mice. In females, alcohol exposure significantly elevated the expression of GRP78 and GRP94 in MANF KO animals at 1 day post gavage (Figures 8A,B), which remained at high levels even after 60 days (Figures 8C,D). The expression of p-IRE1α and XBP1s in MANF KO females were higher than that of control females at 1 day post water gavage; alcohol exposure did not further upregulate them (Figures 8A,B). p-PERK expression was lower in female KO than control, and was significantly reduced by alcohol treatment in MANF KO females at 60 days post gavage (Figures 8C,D). In males, only GRP94 was significantly upregulated in alcohol-exposed MANF KO mice at 1 day post gavage (Figures 8E,F). By 60 days post gavage, both GRP78 and GRP94 were elevated in alcohol-treated MANF KO mice while p-PERK was downregulated (Figures 8G,H). p-IRE1α and XBP1s were not affected by alcohol in male mice at either time points. We further examined if ER stress associated neuronal apoptosis was induced by alcohol treatment or MANF deficiency, but no activation of apoptosis was detected (data not shown). These results suggested that MANF deficiency results in sensitization of alcohol-induced changes of UPR-related markers in a sex-specific manner.

### Effects of binge alcohol exposure and neuronal MANF deficiency on neuroinflammation

**FIGURE 9:**
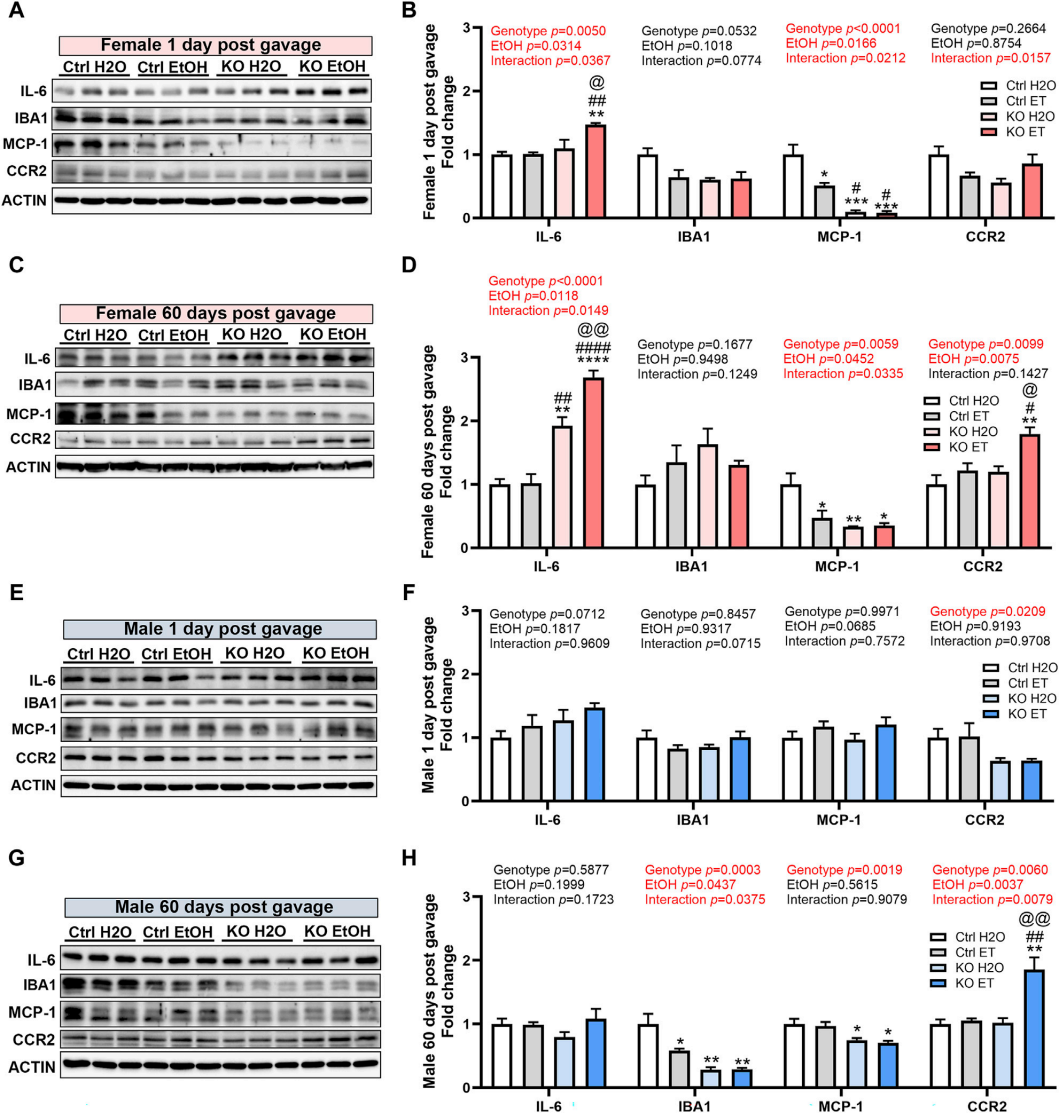
Effects of binge alcohol exposure and neuronal MANF deficiency on neuroinflammation. **(A–D)** Representative immunoblots **(A, C)** and quantification **(B, D)** of neuroinflammation markers in female control and MANF KO cerebral cortex 1 day **(A, B)** or 60 days **(C, D)** post H_2_O or EtOH treatment. **(E–H)** Representative immunoblots **(E, G)** and quantification **(F, H)** of neuroinflammation markers in male control and MANF KO cerebral cortex 1 day **(E, F)** or 60 days **(G, H)** post H_2_O or EtOH treatment. All data were expressed as mean ± SEM. n = 3 per group. Two-way ANOVA followed by Tukey’s *post hoc* test. *p < 0.05, **p < 0.01, ***p < 0.001 when compared to H_2_O treated control. #*p* < 0.05, ##*p* < 0.01, ####*p* < 0.0001 when compared to EtOH treated control. @*p* < 0.05, @@*p* < 0.01 when compared to H_2_O treated KO.

Alcohol exposure can induce neuroinflammation and the activation of microglia, potentially contributing to chronic alcohol-seeking behavior and the behavioral and neurotoxic effects of alcohol (He and Crews, 2008; McClain et al., 2011; Crews and Vetreno, 2016; Crews et al., 2017). MANF has been reported to regulate neuroinflammation and modulate the immune response during tissue repair and regeneration (Neves et al., 2016; Sousa-Victor et al., 2018). We sought to determine the effects of binge alcohol exposure and MANF deficiency on neuroinflammation in the adult brain. We examined the expression of pro-inflammatory cytokine interleukin-6 (IL-6); and markers for microglia activation including ionized calcium binding adaptor molecule 1 (IBA1), monocyte chemoattractant protein-1 (MCP-1) and its receptor C-C motif chemokine receptor 2 (CCR2). In females, IL-6 was not induced by alcohol in control animals. However, it was significantly upregulated in alcohol exposed MANF KO female brain at both 1 day and 60 days post gavage (Figures 9A–D). IBA1 expression in females was not affected by alcohol nor MANF KO. MCP-1 expression was reduced in alcohol-treated female control mice and further downregulated in MANF KO mice at both 1 day and 60 days post gavage (Figures 9A–D). The expression of CCR2 was upregulated in alcohol-treated MANF KO female mice at 60 days post gavage (Figures 9C,D). In males, IL-6 expression was not affected by alcohol nor MANF deficiency (Figures 9E–H). The expression of IBA1 was reduced in alcohol-exposed controls and in MANF KO mice at 60 days post gavage (Figures 9G,H). The expression of MCP-1 was not affected by alcohol but was downregulated in MANF KO mice at 60 days post gavage (Figures 9G,H). The expression of CCR2 was increased in alcohol-exposed MANF KO male mice (Figures 9G,H). These results suggested that there was an intricate neuroimmune response to alcohol and MANF deficiency in a sex-dependent manner.

### Effects of alcohol exposure and neuronal MANF deficiency on the expression of MANF interacting proteins

**FIGURE 10:**
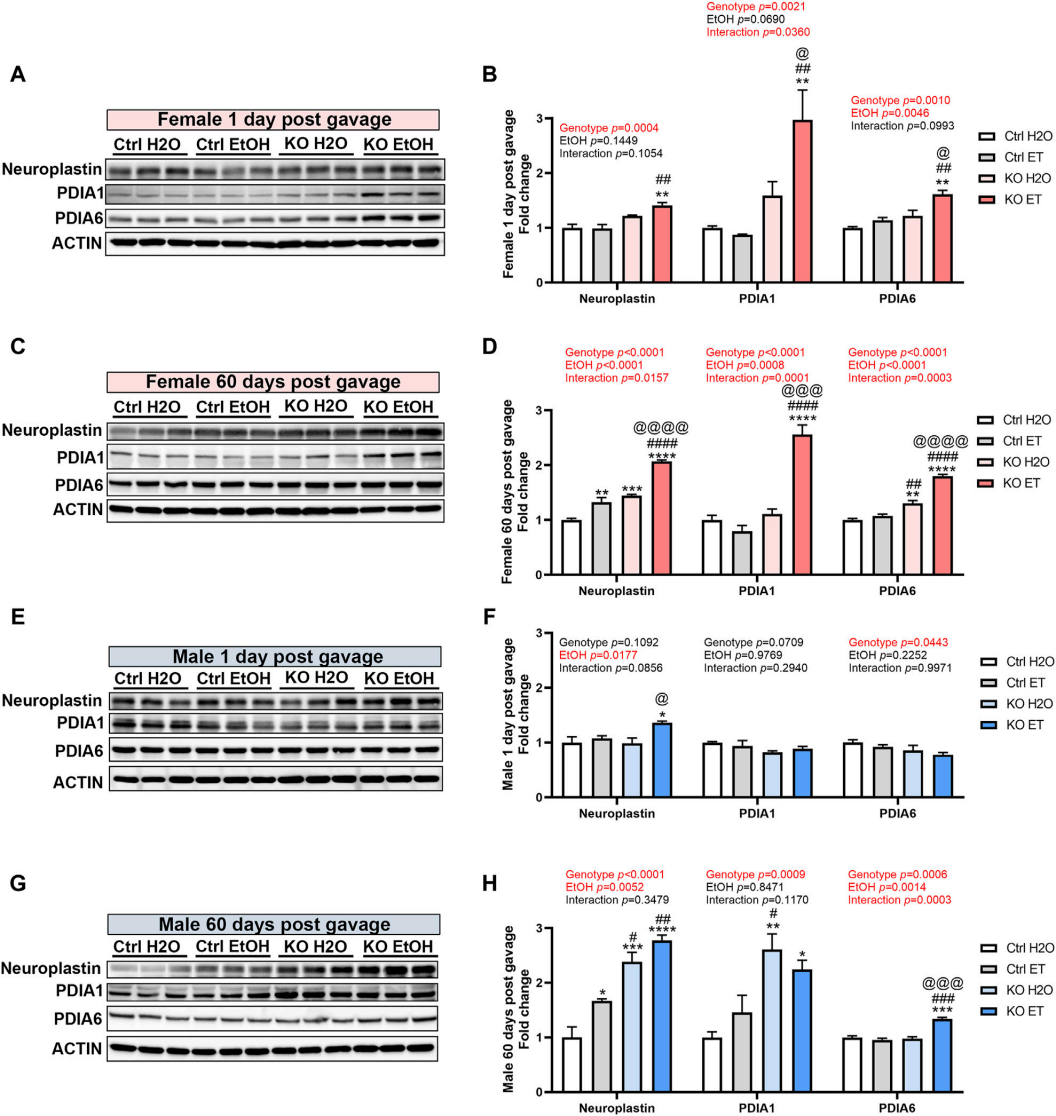
The effect of binge alcohol exposure and neuronal MANF deficiency on the expression of MANF interacting proteins. **(A–D)** Representative immunoblots **(A, C)** and quantification **(B, D)** of MANF interacting proteins neuroplasin, PDIA1, and PDIA6 in female control and MANF KO cerebral cortex 1 day **(A, B)** or 60 days **(C, D)** post H_2_O or EtOH treatment. **(E–H)** Representative immunoblots **(E, G)** and quantification **(F, H)** of MANF interacting proteins neuroplasin, PDIA1, and PDIA6 in male control and MANF KO cerebral cortex 1 day **(E, F)** or 60 days **(G, H)** post H_2_O or EtOH treatment. All data were expressed as mean ± SEM. n = 3 per group. Two-way ANOVA followed by Tukey’s *post hoc* test. *p < 0.05, **p < 0.01, ***p < 0.001, ****p < 0.0001 when compared to H_2_O treated control. #*p* < 0.05, ##*p* < 0.01, ####*p* < 0.0001 when compared to EtOH treated control. @*p* < 0.05, @@*p* < 0.01, @@@*p* < 0.001, @@@@*p* < 0.0001 when compared to H_2_O treated KO.

The action of MANF may be mediated and compensated by its interacting proteins. Neuroplastin and protein disulfide isomerases (PDIs) have been reported as key MANF interacting proteins that exhibit physical binding with MANF (Yagi et al., 2020; Eesmaa et al., 2021). We sought to determine whether alcohol exposure and MANF deficiency affected the expression of neuroplastin, PDIA1, and PDIA6 in the adult brain. In females, the expression of neuroplastin was increased in alcohol-exposed MANF KO mice at 1 day post gavage (Figures 10A,B). At 60 days post gavage, the expression of neuroplastin was increased in alcohol-exposed control mice and further upregulated in alcohol-exposed MANF KO mice (Figures 10C,D). The expression of PDIA1 was upregulated in alcohol-exposed MANF KO female mice at both 1 day and 60 days post gavage (Figures 10A–D). Similarly, the expression of PDIA6 was increased in alcohol-exposed MANF KO female mice at both 1 day and 60 days post gavage (Figures 10A–D). In males, the expression of neuroplastin was increased in alcohol-exposed MANF KO mice at 1 day post gavage (Figures 10E,F). At 60 days post gavage, the expression of neuroplastin was increased in alcohol-exposed control mice and further upregulated in MANF KO mice (Figures 10G,H). The expression of PDIA1 was elevated in male MANF KO mice after at 60 days post gavage (Figures 10G,H). The expression of PDIA6 was upregulated in alcohol-exposed MANF KO male mice at 60 days post gavage (Figures 10E–H).

## Discussion

In this study, we investigated the effects of neuronal MANF deficiency on alcohol-induced neurobehavioral deficits and ER stress in adult mice. We demonstrated that female MANF KO animals were more susceptible to alcohol-induced body weight loss. Female MANF KO mice exhibited increased locomotor activity which was determined by open field test. Barnes maze test showed that learning and memory was not impaired, but it was altered by MANF deficiency in females while it was affected by alcohol treatment in males. For both female and male mice, alcohol exposure and MANF deficiency worked together to increase sociability as determined by the three-chamber sociability task. MANF deficiency sensitized the brain to alcohol-induced alterations of ER stress and neuroinflammation markers in a sex-specific manner. The expression of several key MANF interacting proteins including neuroplastin, PDIA1, and PDIA6 was upregulated in MANF KO mice; their expression was further increased by alcohol exposure. These results demonstrate that alcohol exposure and neuronal MANF deficiency interact to alter neurobehavioral outcomes, ER homeostasis and neuroinflammation in adult mice in a sex-specific manner.

The ER is the largest organelle of the cell that is involved in protein, calcium (Ca^2+^), and lipid homeostasis. In neurons, the ER extends throughout the cell soma and axodendritic compartments and is critical for neuronal functions. A third of the proteome of a cell, secreted and membrane-bound proteins, are processed within the ER lumen and most of these proteins are vital for neuronal activity (Parkkinen et al., 2023). The developing brain is sensitive to alcohol neurotoxicity, and ER stress has been proposed as an important underlying mechanism, which was supported by convincing evidence (Ke et al., 2011; Alimov et al., 2013; Yang and Luo, 2015; Li et al., 2019; Wen et al., 2023). The cellular adaptive system to maintain ER and protein homeostasis, such as UPR and autophagy is developmentally regulated (Alimov et al., 2013). We have previously showed that the sensitivity to alcohol neurotoxicity during the development was inversely correlated with the maturity of stress responsive system including UPR and autophagy (Alimov et al., 2013). In the adult brain, the cellular adaptive system to maintain ER and protein homeostasis has been developed, but the role of ER stress in alcohol neurotoxicity in mature brain is unknown. The current study has provided an important insight into the impact of the interaction of alcohol exposure and ER stress on brain functions in adult mice. In the developing brain, alcohol induced neurodegeneration which was potentiated by MANF deficiency in mice (Wang et al., 2021). The alleviation of ER stress by chemical ER stress inhibitors protected immature neurons against alcohol neurotoxicity, confirming the involvement of ER stress (Li et al., 2019; Wang et al., 2021). Unlike in the developing CNS, binge alcohol exposure did not cause neuroapoptosis and neurodegeneration in the adult brain. However, when the ER homeostasis system is compromised in MANF deficient mice, the mature brain is also impacted as demonstrated by increased neurobehavioral deficits and ER stress/neuroinflammation. It has been shown that neuronal MANF deficient adult mice exhibit chronic UPR activation and were more susceptible to chemical induced ER stress *in vitro* (Pakarinen et al., 2020). Together, the findings of current study with adult mice and those obtained from the developing CNS support a role of ER stress in alcohol neurotoxicity and that the compromised ER homeostasis may exacerbate alcohol’s effects on the brain and subsequent neurobehavioral deficits.

It has been demonstrated in human and mice that binge alcohol exposure could impair learning and memory (Bowden and McCarter, 1993; Sneider et al., 2013; Wagner et al., 2014; Jimenez Chavez et al., 2022). Our results indicated that learning and memory was not significantly affected by alcohol and MANF deficiency. However, a main effect of genotype was detected in female and a main effect of alcohol was detected in male. Currently, it is unknown for the cellular and molecular mechanisms underlying the interaction of alcohol and MANF deficiency, and sex-dependent effects are to be determined. Although neurodegeneration was not observed with the alcohol exposure paradigm in this study, neuronal functions may be impacted by the disruption of ER homeostasis. It is now known that ER is not only involved in stress-mediated neurodegeneration but also axon regrowth, remyelination, neurotransmitter switching, information processing, and regulation of pre- and post-synaptic functions (Khan, 2022). ER might not only be a protein-synthesizing and quality control machinery but also orchestrates neuronal plasticity to execute higher-order brain functions and neural repair (Khan, 2022). More detailed studies including the analysis of neurogenesis, neurotransmitter levels, axonal/dendritic networks, and synaptic functions on focused brain regions, such as hippocampus, are necessary in the future. Further study using chemical ER stress inhibitors and genetic manipulations may provide more distinct interpretation.

Our study showed that binge alcohol exposure and MANF deficiency interacted to enhance sociability. It has been reported in human and mice that binge alcohol drinking increased sociability (Gmel et al., 2020; Kersey et al., 2022; Simon et al., 2023). The cellular/molecular mechanisms underlying alcohol-enhanced sociability are not fully elucidated, so is the role of MANF in this process. One possible mechanism could involve the interaction between alcohol and anxiety/stress pathways. Alcohol has been shown to affect the amygdala, a brain region involved in emotional processing, and the hypothalamic-pituitary-adrenal (HPA) axis, which regulates the stress response (Silberman et al., 2009; Heinz et al., 2011). Functional changes in these brain regions could impact social behavior and sociability. MANF is widely expressed in these brain regions. It is of interest to investigate how alcohol exposure and MANF deficiency interact to influence the function of these brain regions.

Another important finding of the current study is that sex difference plays a role in alcohol induced neurobehavior and molecular changes. We observed sex-specific responses to alcohol exposure and MANF deficiency on body/brain weight, some neurobehavioral outcomes, and the expression of ER stress and neuroinflammation markers. It has been suggested that sex differences may play a critical role in modulating how alcohol exposure impacts the brain and the development of alcohol use disorder (AUD) (Wilsnack et al., 2018; Peltier et al., 2019; Fama et al., 2020; Finn, 2020; Flores-Bonilla and Richardson, 2020; White, 2020; Hitzemann et al., 2022). In the past, rates of AUD were higher in men than in women, but over the past 10 years, the difference between sexes in prevalence of AUD has narrowed because of a much higher increased rate of female AUD; Over the last 10 years, rates of AUD have increased in women by 84% relative to a 35% increase in men (Verplaetse et al., 2021). Although heavy drinking affects working memory, visuospatial abilities, balance, emotional processing, and social cognition in both women and men, sex differences mark the severity and specific profile of functional deficits. Research suggests that, although women tend to drink less than men, a risk-severity paradox occurs wherein women suffer greater harms than men at lower levels of alcohol exposure (Foster et al., 2018). In experimental studies, alcohol’s effects on the mouse brain are modulated by sex with female mice showing greater loss of brain volume than male mice (Piekarski et al., 2022). Recent studies also suggest that females are more susceptible than males to alcohol-induced liver inflammation, cardiovascular disease, memory blackouts, hangovers, and certain cancers (White, 2020). However, research often has not given close attention to sex differences in the etiology and the consequences of AUD. Our results suggest that there may be a sex-specific susceptibility to alcohol-induced ER stress/neuroinflammation in the brain, which may contribute to pathological and neurobehavioral outcomes. Therefore, investigation of molecular mechanisms underlying sex differences in alcohol-induced ER stress/inflammation in the brain may provide critical insight into the sex-specific susceptibility. Sex dimorphism of ER stress has been reported in alcohol-induced liver injury mouse model (Walter et al., 2024). Sex differences in alcohol-induced neuroinflammation have also been reported in an intermittent alcohol binge drinking mouse model, with increased several cytokines, including IL-17A and IL-1β, and chemokines, such as MCP-1 and MIP-1α in the prefrontal cortex and serum of adolescent female mice but not in males, suggesting that female mice are more vulnerable than males to the inflammatory effects of binge ethanol drinking (Pascual et al., 2017). In our future studies, we plan to use new spatial RNA mapping technology, such as 10× Visium Spatial Transcriptomics, MERSCOPE and Xenium to identify differentially expressed genes in the male and female brain in response to alcohol exposure in MANF KO and their littermates. Considering sex-specific molecular changes is important in understanding the impact of alcohol on neuronal functions and critical for the development of more effective prevention/therapeutic strategies, because therapeutic strategies based on the research on men may not be effective in women.

The molecular pathways through which MANF execute its neurotrophic function is poorly understood. Several MANF interacting proteins have been identified that may be involved MANF’s function and phenotypes associated with MANF deficiency. MANF physically binds with several ER-resident protein disulfide isomerases (PDIs) including PDIA1 and PDIA6 (Eesmaa et al., 2021). PDIs are enzymes that catalyze disulfide bond formation, isomerization, and reduction, crucial for protein folding and function. In our study, PDIA1 and PDIA6 were both upregulated in the brain of alcohol treated MANF KO animals. Similar upregulation of the PDIs was observed in a cardiomyocyte-specific MANF-knockdown mouse model in response to reductive stress (Arrieta et al., 2020). This evidence suggests that there is potential crosstalk and functional compensation between MANF and PDIs, and MANF may maintain ER homeostasis by cooperate with PDIs to modulate protein folding and maturation in the ER. MANF has been shown to bind to a cell surface receptor neuroplastin to regulate inflammatory responses and cell death (Yagi et al., 2020). The binding of MANF to neuroplastin has been suggested to reduce inflammation and apoptosis by inhibiting NF-κB signaling, indicating a potential anti-inflammatory effect mediated by this interaction (Yagi et al., 2020). The increased expression of neuroplasin in MANF KO brains and its further upregulation by alcohol treatment may be a compensatory response to MANF deficiency. Further studies into the downstream targets of the MANF-neuroplastin complex could provide valuable insights into alcohol induced neuroinflammation.

In conclusion, alcohol exposure and neuronal MANF deficiency interacted to alter neurobehavioral outcomes, ER homeostasis and neuroinflammation in adult mice in a sex-specific manner. It is important to develop strategies for targeted intervention and therapy for excessive alcohol consumption associated neuronal damage. For example, environmental enrichments or specific chemicals were found effective to mitigate alcohol-induced cognitive deficits and drinking behavior (Brancato et al., 2020; Tringali et al., 2023). Our findings demonstrate that MANF and ER stress may play a role in alcohol-induced neurobehavioral deficits, suggesting that they are potential targets for developing intervention strategies. Understanding the sex-specific responses to alcohol neurotoxicity is crucial for developing targeted interventions and treatment strategies that consider the unique vulnerabilities and responses of males and females to alcohol-induced damage. Further research into the molecular mechanisms underlying these sex differences is essential for advancing our knowledge of alcohol neurotoxicity.

## Data Availability

The original contributions presented in the study are included in the article/Supplementary Material, further inquiries can be directed to the corresponding author.
